# Discovery of ammosesters by mining the *Streptomyces uncialis* DCA2648 genome revealing new insight into ammosamide biosynthesis

**DOI:** 10.1093/jimb/kuab027

**Published:** 2021-03-24

**Authors:** Jun Luo, Dong Yang, Hindra, Ajeeth Adhikari, Liao-Bin Dong, Fei Ye, Xiaohui Yan, Christoph Rader, Ben Shen

**Affiliations:** Department of Chemistry, The Scripps Research Institute, Jupiter, FL 33458, USA; Department of Chemistry, The Scripps Research Institute, Jupiter, FL 33458, USA; Natural Products Discovery Center at Scripps Research, The Scripps Research Institute, Jupiter, FL 33458, USA; Department of Chemistry, The Scripps Research Institute, Jupiter, FL 33458, USA; Department of Chemistry, The Scripps Research Institute, Jupiter, FL 33458, USA; Department of Immunology and Microbiology, The Scripps Research Institute, Jupiter, FL 33458, USA; Department of Chemistry, The Scripps Research Institute, Jupiter, FL 33458, USA; Department of Chemistry, The Scripps Research Institute, Jupiter, FL 33458, USA; Department of Chemistry, The Scripps Research Institute, Jupiter, FL 33458, USA; Department of Immunology and Microbiology, The Scripps Research Institute, Jupiter, FL 33458, USA; Department of Chemistry, The Scripps Research Institute, Jupiter, FL 33458, USA; Natural Products Discovery Center at Scripps Research, The Scripps Research Institute, Jupiter, FL 33458, USA; Department of Molecular Medicine, The Scripps Research Institute, Jupiter, FL 33458, USA

**Keywords:** Ammosester, Ammosamide, Genome mining, *Streptomyces uncialis*, Biosynthesis

## Abstract

The ammosamides (AMMs) are a family of pyrroloquinoline alkaloids that exhibits a wide variety of bioactivities. A biosynthetic gene cluster (BGC) that is highly homologous in both gene content and genetic organization to the *amm* BGC was identified by mining the *Streptomyces uncialis* DCA2648 genome, leading to the discovery of a sub-family of new AMM congeners, named ammosesters (AMEs). The AMEs feature a C-4a methyl ester, differing from the C-4a amide functional group characteristic to AMMs, and exhibit modest cytotoxicity against a broad spectrum of human cancer cell lines, expanding the structure–activity relationship for the pyrroloquinoline family of natural products. Comparative analysis of the *ame* and *amm* BGCs supports the use of a scaffold peptide as an emerging paradigm for the biosynthesis of the pyrroloquinoline family of natural products. AME and AMM biosynthesis diverges from a common intermediate by evolving the pathway-specific Ame24 *O*-methyltransferase and Amm20 amide synthetase, respectively. These findings will surely inspire future efforts to mimic Nature's combinatorial biosynthetic strategies for natural product structural diversity.

## Introduction

**Fig. 1.**
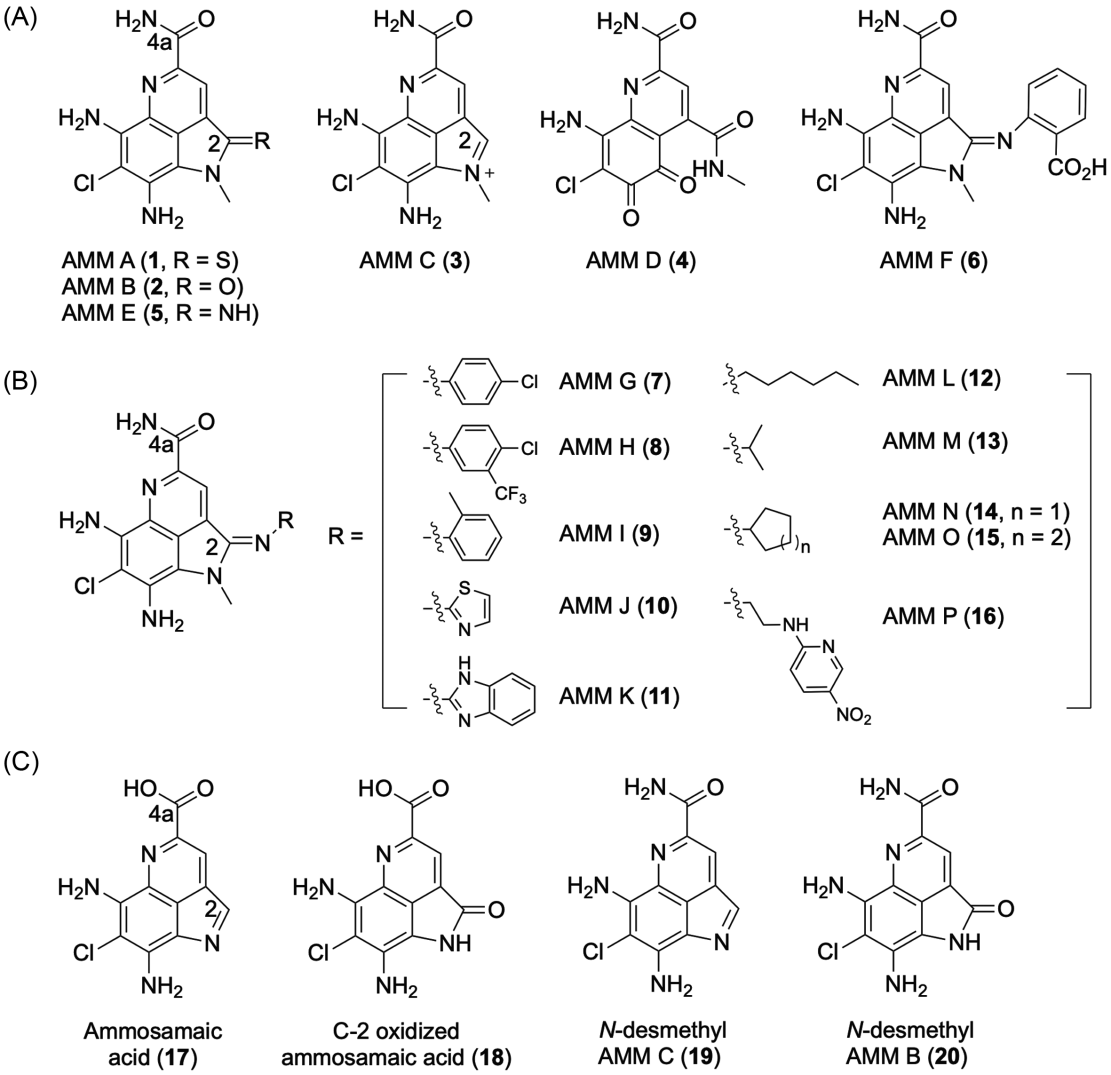
Structures of known ammosamides (AMMs). (A) Naturally isolated AMMs: **1–3** were produced by *Streptomyces* sp. CNR-698 wild-type strain and **4–6** were produced by *S. variabilis* SAN-020 wild-type strain. (B) Amidine analogs of AMMs: **7–16** were produced by precursor-directed biosynthesis in *S. variabilis* SAN-020 wild-type strain. (C) Engineered AMMs, and ammosamaic acid and congener: **17–20** were produced by *Streptomyces* sp. CNR-698 mutant strains.

The ammosamides (AMMs) are a family of biologically active natural products featuring an unusual chlorinated pyrrolo[4,3,2-*de*]quinoline core (Fig. 1). The first discovered members, AMMs A (**1**) and B (**2**), were isolated from a marine-derived strain *Streptomyces* sp. CNR-698 (Fig. 1A) (Hughes, MacMillan, Gaudêncio, Fenical, et al., 2009; Hughes, MacMillan, Gaudêncio, Jensen, et al., 2009). Subsequent efforts in the total syntheses of **1** and **2** and generation of additional analogs for structure–activity relationship (SAR) studies enabled the discovery of AMM C (**3**), the third member of the AMM family from *Streptomyces* sp. CNR-698 and the likely biosynthetic precursor to **1** and **2** upon nucleophilic oxidation or sulfuration at C-2, respectively (Fig. 1A) (Hughes & Fenical, 2010). Since then, an oxidatively ring-opened analog AMM D (**4**) and two amidine analogs AMMs E (**5**) and F (**6**) have been isolated from another marine-derived strain *Streptomyces variabilis* SAN-020, together with **1** and **2** (Fig. 1A) (Pan et al., 2012, 2013). Supplementing the culture medium of *S. variabilis* SAN-020 with a variety of aryl and alkylamines further afforded the production of AMMs G-P (**7**–**16**) (Fig. 1B) (Pan et al., 2013). Subsequently, **3** was established as the only bona fide natural product of the AMM biosynthetic machinery, and all other AMMs isolated to date were “artifacts,” derived from **3** via a nonenzymatic nucleophilic addition of the varying nucleophiles at C-2 followed by air oxidation, during fermentation and isolation of the natural products (Reimer & Hughes, 2017). While **1** and **2** were initially shown to be highly cytotoxic against the HCT-116 colon cell line, targeting the motor protein myosin (Hughes, MacMillan, Gaudêncio, Fenical, et al., 2009; Hughes, MacMillan, Gaudêncio, Jensen, et al., 2009), **2** and several of its synthetic analog have been shown to be potent inhibitors against quinone reductase 2, a cytosolic protein that has been implicated as a target for cancer chemoprevention (Reddy et al., 2012; Reimer & Hughes, 2017). Discovery of novel AMM congeners could therefore enrich the structural diversity around this privileged scaffold and expand the SAR of AMMs for potential drug discovery.

**Fig. 2.**
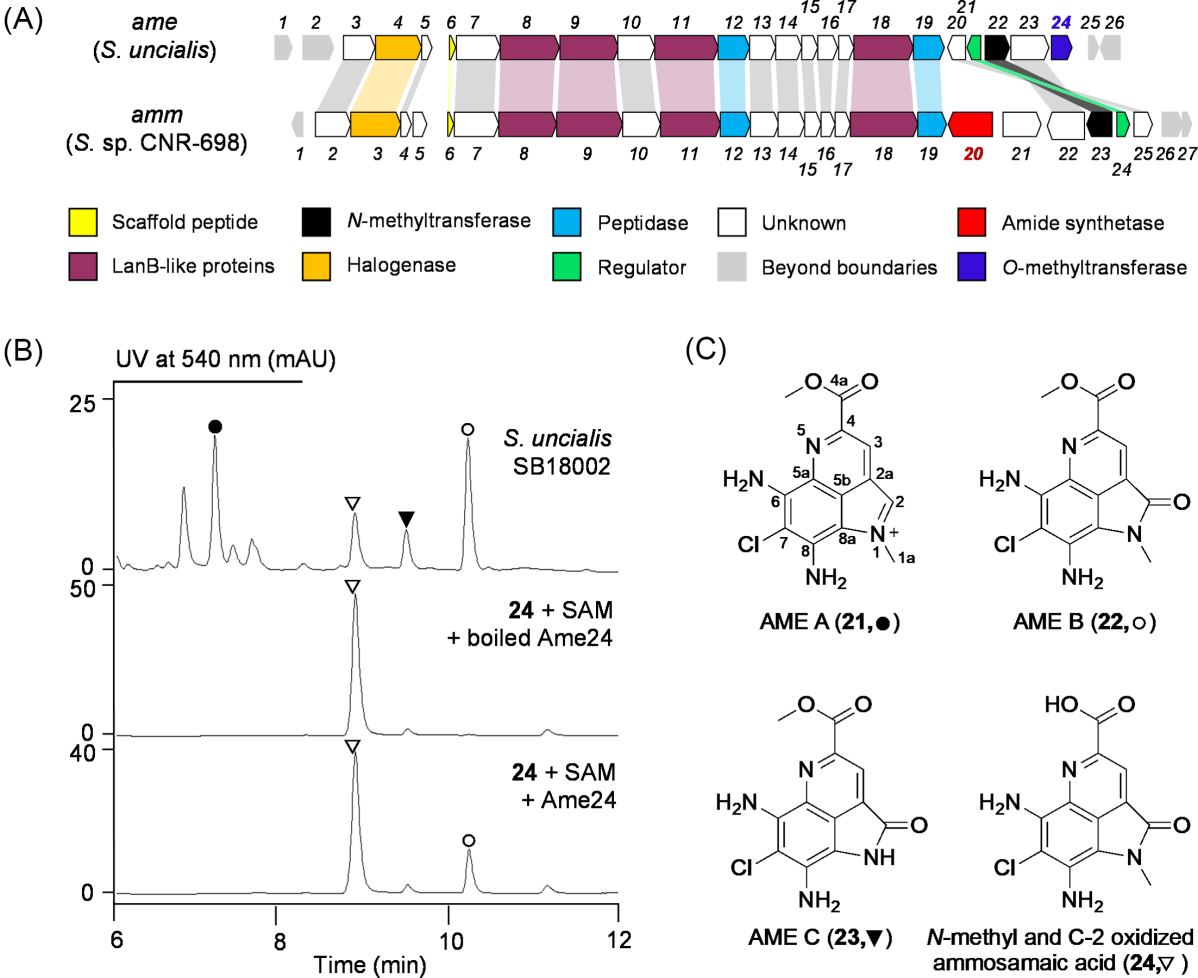
Genome mining and characterization of *S. uncialis* as an ammosester producer. (A) Genetic organization of the *ame* and *amm* BGCs. Functional annotations of the *ame* and *amm* BGCs are summarized in Supplementary Table S3, and genes common to both BGCs are color-shaded and cross-shaded. The pathway specific Ame24 *O*-methyl transferase and Amm20 amide synthetase are color-coded in blue and red, respectively. (B) HPLC analysis of metabolite profile of *S. uncialis* SB18002 fermentation and *in vitro* assays of Ame24-catalyzed C-4a *O*-methylation of **24** in the presence of SAM, affording **22**. (C) Structures of AMEs A (**21**), B (**22**), C (**23**), and the ammosamaic acid congener (**24**) from *S. uncialis* SB18002.

The ammosamide (*amm*) biosynthetic gene cluster (BGC) has been cloned from *Streptomyces* sp. CNR-698 (Fig. 2A) and expressed in *Streptomyces coelicolor* M512, leading to the production of **1**–**3** and providing a genetically amenable platform to interrogate AMM biosynthesis *in vivo* (Jordan & Moore, 2016). The deceptively simple AMM scaffold is of a surprisingly complex biosynthetic origin, as the *amm* BGC encodes a precursor peptide (Amm6) and LanB-like proteins (such as Amm8, 9, 11, and 18), only known to be associated with ribosomally synthesized and posttranslationally modified peptides (RiPPs) at the time (Arnison et al., 2013). Deletion of the genes encoding either Amm6 or the LanB-like proteins yielded mutants that completely lost AMM production, confirming their necessity for AMM production but failing to reveal any insight into their roles in AMM biosynthesis (Jordan & Moore, 2016). In contrast, deletion of the genes encoding selected tailoring enzymes, such as a F420-dependent oxidase (Amm4) and an *N*-methyltransferase (Amm23), afforded mutants that accumulated ammosamaic acid (**17**) and *N*-desmethyl AMM C (**19**), as well as their corresponding air-oxidized (at C-2) congeners (**18** and **20**), shedding light on the late steps of AMM biosynthesis (Jordan & Moore, 2016). Most recently, it has been discovered that the AMMs belong to an emerging family of amino acid-derived natural products whose biosynthesis features a ribosomally synthesized scaffold peptide that undergoes a nonribosomal peptide extension, catalyzed by the LanB-like proteins, followed by tailoring modifications of the extended amino acid and an eventual proteolytic removal of the scaffold peptide to afford the final natural products (Ting et al., 2019). This was demonstrated for AMMs by *in vitro* extension of an Amm6 (also known as AmmA) variant with L-Trp, catalyzed by Amm9 (also known as AmmB2) (Ting et al., 2019). Pending experimental confirmation, tailoring modifications of the extended L-Trp followed by proteolytic removal of the Amm6 scaffold peptide would yield a biosynthetic intermediate featuring the pyrroloquinoline core with a free carboxylic acid, in agreement with the isolation of **17** and **18** (Fig. 1C) (Jordan & Moore, 2016). Comparison of the BGCs encoding AMMs and novel congeners therefore promises to reveal new insights into the use of a scaffold peptide in the biosynthesis of amino acid-derived natural products and provide new opportunities to mimic Nature's combinatorial biosynthetic strategies to generate natural product structural diversity.

**Fig. 3.**
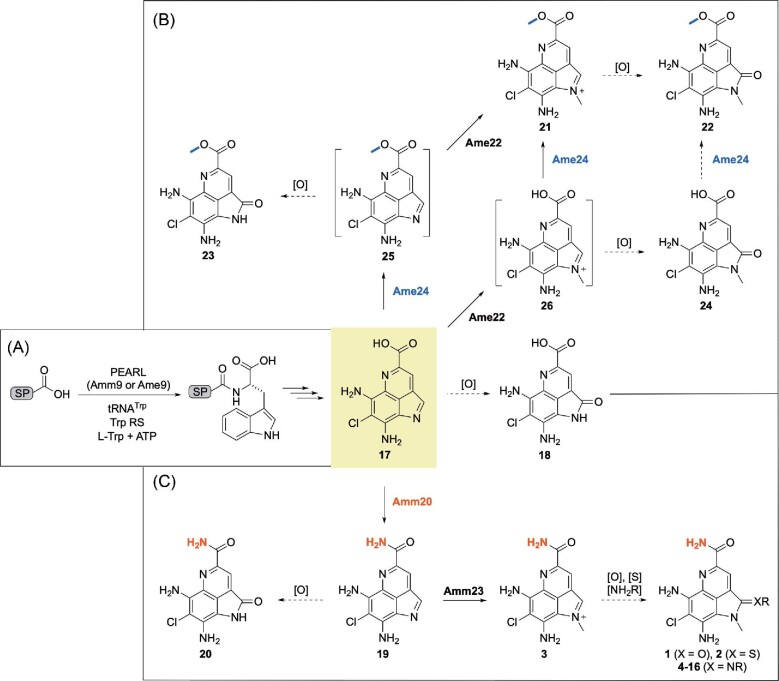
A unified proposed pathway for ammosester and ammosamide biosynthesis diverging from a common intermediate with pathway-specific tailoring enzymes. (A) The use of a scaffold peptide as an emerging paradigm for the biosynthesis of the pyrroloquinoline family of natural products. (B) AME and (C) AMM biosynthesis diverges from the common intermediate **17** by evolving the pathway-specific Ame24 *O*-methyltransferase (color-coded blue) and Amm20 amide synthetase (color-code red), respectively. The structures in brackets represent proposed intermediates; bold arrows represent the proposed major routes and dashed arrows depict nonenzymatic paths or enzyme substrate promiscuity. PEARL, peptide-amino acid acyl tRNA ligase (Ting et al., 2019); SP, scaffold peptide; [O], oxidation; [S], sulfuration; [NH_2_R], aryl and alkylamines.

*Streptomyces uncialis* DCA2648, a lichen-associated actinomycete, is known to produce uncialamycin (UCM) (Davies et al., 2005), a member of the enediyne family of extremely potent antitumor antibiotics (Adhikari et al., 2020), and the cladoniamides (CLAs) (Williams et al., 2008), bis-indole alkaloids with modest cytotoxicity against selected human cancer cell lines. The *cla* BGC was cloned previously by screening a cosmid library of *S. uncialis* (Ryan 2011). We cloned the *ucm* BGC by sequencing and mining the *S. uncialis* genome for genes encoding the characteristic enediyne polyketide synthase cassette (Yan et al., 2016). Genome mining has revolutionized microbial natural product discovery in the genomics era (Kalkreuter et al., 2020; Ziemert et al., 2016). We have applied multiple strategies in our current efforts to leverage the large strain collection at The Scripps Research Institute for natural product and drug discovery by genome mining (Steele et al., 2019), as exemplified by the discovery of new enediynes (Yan et al., 2016; Yan et al., 2017) and the leinamycin family of natural products (Pan et al., 2017). The *S. uncialis* genome harbors minimally 30 BGCs (Yan et al., 2016), providing an outstanding opportunity to mine for novel natural products. Here we report the discovery of the ammosesters (AMEs), a sub-family of new AMM congeners that feature a C-4a methyl ester (Fig. 2), differing from the C-4a amide functional group characteristic to the AMMs (Fig. 1), from *S. uncialis* DCA2648. Comparative analysis of the *amm* and *ame* BGCs supports the use of a scaffold peptide in the biosynthesis of the common pyrroloquinoline carboxylic acid intermediate **17**, from which AME and AMM biosynthesis diverges by evolving a pathway-specific *O*-methyltransferase (Ame24) for AMEs and amide synthetase (Amm20) for AMMs, respectively (Fig. 3). The AMEs exhibit modest cytotoxicity against selected human cancer cell lines, further expanding the SAR for the pyrroloquinoline family of natural products.

## Materials and Methods

 

### Bacterial Strains and Culture Conditions

Bacterial strains, plasmids, and oligonucleotides used in this study are listed in Supplementary Tables S1 and S2. The cosmid library of *S. uncialis* was constructed previously (Yan et al., 2016). *Escherichia coli* strains were grown in lysogeny broth (LB) with appropriate antibiotic selection (Sambrook & Russel, 2001). PCR primers were obtained from Sigma-Aldrich. *S. uncialis* strains were cultured using a two-stage fermentation. After growth on the ISP-4 plate at 28°C for 10–14 days, the spores of *S. uncialis* strains were collected and cultured in 250-ml baffled flasks containing 50 ml of MYM medium (maltose 4 g/l, yeast extract 4 g/l, malt extract 10 g/l, pH 7.0) at 28°C and 250 rpm for 2 days. The seed culture (5 ml) was then transferred into 250-ml baffled flasks containing 50-ml production medium [soluble starch 10 g/l, malt extract 2.5 g/l, K_2_HPO_4_ 1 g/l, MgSO_4_·7H_2_O 1 g/l, (NH_4_)_2_SO_4_ 2 g/l, CaCO_3_ 2 g/l, CuSO_4_·5H_2_O 0.01 g/l, KI 0.005 g/l, pH 7.0] and cultured at 28°C and 250 rpm for 10 days.

### Experimental Procedures

Bioinformatics analyses and comparative analyses of nucleotide or amino acid sequences were conducted using BLAST. Fermentation was carried out in New Brunswick Scientific Innova 44 incubator shakers. The ^1^H and ^13^C, HSQC, and HMBC spectra were collected with a Bruker Avance III Ultrashield 700 at 700 MHz for ^1^H and 175 MHz for ^13^C nuclei. HPLC was carried out on an Agilent 1260 Infinity LC equipped with an Agilent SB-C18 column (250 mm × 4.6 mm, 5 µm) at a flow rate of 1 ml/min. The column was equilibrated with 10% acetonitrile with 0.1% formic acid, and developed with a 20-min analytical program consisting of a 15-min linear gradient from 10% to 70% acetonitrile with 0.1% formic acid followed by a 5-min linear gradient from 70% to 100% acetonitrile with 0.1% formic acid. LC–MS was performed on an Agilent 1260 Infinity LC coupled to a 6230 TOF (HR-ESI) equipped with an Agilent Poroshell 120 EC-C18 column (50 mm × 4.6 mm, 2.7 µm) using a linear gradient of CH_3_CN in H_2_O with 0.1% formic acid (0–15 min, 10–70% CH_3_CN; 15–20 min, 70–100% CH_3_CN) at a flow rate of 0.4 ml/min. Column chromatography was conducted on Sephadex LH-20. Semipreparative HPLC was carried out on a Varian liquid chromatography system with a YMC-pack ODS-A (250 mm × 10 mm, 5 µm) column. All common biochemicals and culture medium components were purchased from commercial sources and used as it.

### Extraction and Isolation

After the fermentation, 1% Amberlite XAD-16 and 1% Dianion HP-20 resins were added to the fermentation broth and incubated overnight at 28°C. The cell pellets and resins were collected by centrifuge, dried on air, and then extracted three times with methanol. The extract was concentrated and subjected to HPLC or LC–MS for analysis. For large-scale fermentation, 2-l baffled flasks each containing 400 ml of production medium were inoculated with 40 ml of the seed culture and cultured at 28°C on a rotary shaker at 250 rpm for 10 days. The fermentation broth (10 l) was extracted as described above. The extracts were dried in vacuo and subjected to Sephadex LH-20 and semipreparative HPLC for isolation to afford compounds **21** (5 mg), **22** (12 mg), **23** (3 mg), and **24** (1 mg), respectively.

*AME A (* ***21*** *), AME B (* ***22*** *), AME C* (***23****), and N-methyl and C-2 oxidized ammosamaic acid (****24***): ^1^H NMR (700 MHz), ^13^C NMR (175 MHz), HR-ESI-MS and UV data, see Table 1 and Supplementary Figs. S1–S5; ***21***, purplish red solid, HR-ESI-MS *m/z* 291.0632 [M]^+^ (calcd for C_13_H_12_ClN_4_O_2_^+^, 291.0643); ***22***, purple powder, HR-ESI-MS *m/z* 307.0598 [M + H]^+^ (calcd for C_13_H_11_ClN_4_O_3_, 307.0592); ***23***, purple powder, HR-ESI-MS *m/z* 293.0448 [M + H]^+^ (calcd for C_12_H_9_ClN_4_O_3_, 293.0436); ***24***, purple powder, HR-ESI-MS *m/z* 293.0444 [M + H]^+^ (calcd for C_12_H_9_ClN_4_O_3_, 293.0436).

**Table 1.** NMR Spectroscopic Data (^1^H at 700 MHz and ^13^C at 175 MHz, DMSO-*d*_6_) for AMEs (**21**–**23**) and the Ammosamaic Acid Congener **24**

	AME A (**21**)		AME B (**22**)		AME C (**23**)		Ammosamaic acid congener (**24**)	
Position	*δ*_C_, type ^a^	*δ*_H_ (*J* in Hz)	*δ*_C_, type	*δ*_H_ (*J* in Hz)	*δ*_C_, type	*δ*_H_ (*J* in Hz)	*δ*_C_, type	*δ*_H_ (*J* in Hz)
1a	38.4, CH_3_	4.34, s	29.1, CH_3_	3.61, s			29.1, CH_3_	3.60, s
2	124.3, C	7.95, s	164.1, C		165.2, C		164.4, C	
2a	121.5, C		130.7, C		131.9, C		131.1, C	
3	121.0, CH	8.48, s	119.0, CH	8.38, s	119.3, CH	8.36, s	118.1, CH	8.35, s
4	138.6, C		142.1, C		141.9, C		ND^b^	
4a	166.5, C		165.8, C		165.9, C		166.7, C	
5a	142.0, C		133.6, C		132.8, C		132.9, C	
5b	119.3, C		119.6, C		120.4, C		119.4, C	
6	142.9, C		132.4, C		132.7, C		131.5, C	
7	106.4, C		105.9, C		105.1, C		105.4, C	
8	154.3, C		140.9, C		140.5, C		140.8, C	
8a	119.1, C		107.0, C		105.2, C		107.1, C	
1N-H						10.22, s		
4a-OCH_3_	52.7, CH_3_	3.90, s	53.0, CH_3_	3.96, s	53.0, CH_3_	3.95, s		
6-NH_2_		8.31, s		6.18, s		6.08, s		6.20, s
8-NH_2_		6.34, s		6.35, s		6.37, s		6.50, s

^a^Carbon chemical shifts were based on HSQC and HMBC data.

^b^No carbon signal was detected, which was consistent with reported ^13^C NMR spectrum for **18** (Jordan & Moore, 2016).

### Cytotoxicity Assay

The EC_50_s of AMEs (**21–23**), together with the ammosamaic acid congener **24**, against a panel of human cancer cell lines, including melanoma (SK-MEL-5), breast (MDA-MB-231), central nervous system (SF-295), ovarian (OVCAR-3), and nonsmall-cell lung cancer (NCI-H226) were determined using the Cell-Titer 96^®^ AQueous One Solution Proliferation Assay (MTS) kit (Promega), according to the manufacturer's suggested protocol (Yan et al., 2016; Yan et al., 2017). AMM B (**2**), obtained by treatment of **22** with ammonia in THF (Reddy et al., 2010), and doxorubicin were used as positive controls. Each point represents the mean ± SD of three replicates, and the EC_50_ was determined by computerized curve fitting using GraphPad Prism (Table 2 and Supplementary Fig. S6).

**Table 2.** Cytotoxicity of AMEs (**21–23**) and the Ammosamaic Acid Congener **24** Against Selected Human Cancer Cell Lines

		EC_50_ (µM)					
Cell lines	Cancer type	**2**	**21**	**22**	**23**	**24**	Doxorubicin
MDA-MB-231	Breast	8.5 ± 2.3	56 ± 22	>200	>200	>200	0.2 ± 0.8
SK-MEL-5	Melanoma	9.3 ± 0.9	21 ± 1	28 ± 8	>200	>200	0.5 ± 0.3
SF-295	CNS	126 ± 20	140 ± 8	>200	>200	>200	4.8 ± 1.1
NCI-H226	NCS lung	80 ± 4	15 ± 7	>200	>200	>200	3.0 ± 0.3
OVCAR-3	Ovarian	8.4 ± 1.1	17 ± 1	47 ± 12	>200	>200	1.4 ± 0.1

### Construction of the Δ*ame24* Mutant Strain *S. uncialis* SB18003

Inactivation of the *ame24* gene was performed by gene replacement following literature procedures (Supplementary Fig. S7) (Gust et al., 2003; Kieser et al., 2000). Briefly, pBS18005, a cosmid containing a partial *ame* gene cluster, was transformed into *E. coli* BW25113/pIJ790. The *ame24* gene was replaced with the apramycin resistance cassette from pIJ773 using λ-RED-mediated PCR-targeting strategy to afford pBS18006. Then pBS18006 was introduced into *S. uncialis* SB18002 by intergeneric conjugation. Exconjugants with the desired double crossover recombination event were selected based on resistance to apramycin and sensitivity to kanamycin, yielding the Δ*ame24* mutant strain SB18003. The genotype of SB18003 was confirmed by Southern blot analysis using DIG High Prime DNA Labeling and Detection Starter Kit I (Roche) (Supplementary Fig. S7). DNA probe was PCR-amplified using oligonucleotides SA-ame24-F/SA-ame24-R (Supplementary Table S2) and *S. uncialis* SB18002 genomic DNA as template.

### Gene Expression and Protein Overproduction and Purification

The *ame24* gene was amplified by PCR from the cosmid pBS18005 using the primers GE-ame24-F/GE-ame24-R (Supplementary Table S2) and Q5 High-Fidelity DNA Polymerase (NEB) following the protocol provided by the manufacturer. The PCR product was purified, treated with T4 polymerase (NEB), and cloned into pBS3080 according to ligation-independent procedures to give pBS18007 (Lohman et al., 2013). pBS18007 was transformed into *E. coli* BL21 (DE3), and cells were grown in LB at 37°C until an OD_600_ of 0.6 was reached. The cells were cooled to 4°C, added 0.1 mM of isopropyl β-d-1-thiogalactopyranoside to induce gene expression, and further grown at 18°C for 16 hr. The cells were harvested at 4000 × *g* for 15 min at 4°C, and the pellet was resuspended in lysis buffer (100-mM Tris, pH 8.0, containing 300-mM NaCl, 15-mM imidazole, and 10% glycerol). After sonication, the cell debris was removed by centrifugation at 15 000 × *g* for 15 min at 4°C. The lysate was loaded onto a HisTrap 5-ml column equilibrated with washing buffer (50-mM Tris, pH 8.0, containing 100-mM NaCl and 15-mM imidazole). The column was washed with washing buffer and the His6-tagged protein was eluted using elution buffer (50-mM Tris, pH 8.0, containing 100-mM NaCl and 300-mM imidazole). Following elution, the protein was diluted three times using 50-mM Tris, pH 8.0 buffer. The protein was loaded onto a HiTrap Q HP 5 ml equilibrated with washing buffer (50-mM Tris, pH 8.0). The column was washed with washing buffer and the protein was eluted using a gradient of elution buffer (50-mM Tris, pH 8.0, containing 1-M NaCl). Finally, the protein was loaded onto a Superdex S200 16/600 gel filtration column using 50-mM Tris, pH 8.0 buffer containing 100-mM NaCl, affording 9.7 mg of Ame24 to homogeneity from 0.8 l of *E. coli* recombinant culture, and stored at −80°C (Supplementary Fig. S8A).

### *In vitro* Assay of Ame24 as a C-4a *O*-Methyltransferase

Enzymatic reactions were performed in 50-mM Tris buffer, pH 7.5, containing 200 μM of Ame24, 500-μM SAM, and 200 μM **24**, in a total volume of 50 μl. After incubation at 30°C for different times, 50 μl of methanol were added to quench the reactions. The reaction mixture was then centrifuged and 10 μl of the supernatant were injected and analyzed by analytical HPLC (Supplementary Fig. S8C).

## Results and Discussion

 

### Identification of a Putative BGC Encoding Ammosamide Family of Natural Products Through Genome Mining of *S. uncialis* DCA2648

*In silico* analysis of the *S. uncialis* genome by antiSMASH (Blin et al., 2019) led to the identification of the *ame* BGC that has a nearly identical genetic organization to the *amm* BGC from *Streptomyces* sp. CNR-698 (Fig. 2A). Comparative analysis of the gene products within the two BGCs revealed high amino acid sequence identity (52–76%), indicative of homologous biosynthetic machinery (Supplementary Table S3). While the *ame* and *amm* BGCs consist of 22 and 24 open reading frames (orfs), respectively, 21 of them are conserved between the two BGCs. Notably, the homolog of *amm20*, which is predicted to encode an amide synthetase, is missing in the *ame* BGC, together with *amm21*, encoding a transporter, and *amm5*, encoding a hypothetical protein, while the *ame* BGC harbors an extra gene, *ame24* encoding an *O*-methyltransferase, the homolog of which is absent in the *amm* BGC (Fig. 2A). The similarity and difference between the two BCGs suggest that *S. uncialis* may produce novel congeners of the AMM family of natural products.

### Production, Isolation, and Structural Elucidation of Ammosesters from *S. uncialis* SB18002

To determine whether *S. uncialis* produces AMM congeners, we initially carried out fermentation of the wild-type *S. uncialis* DCA2648 on a small scale (50 ml). However, detection of new metabolites was obscured by the CLAs, which were the predominate natural products under the optimized fermentation condition. To facilitate detection of the trace metabolites overshadowed by CLAs, we deleted the *cla* BGC in the wild-type strain to afford the Δ*cla* mutant strain of *S. uncialis* SB18002. *S. uncialis* SB18002 was fermented and the fermentation broth was extracted and subjected to HPLC and LC-MS analysis, resulting in identification of several metabolites with a UV absorbance of 520–540 nm and MS isotope signatures of chlorine-containing natural products, characteristic of the AMMs (Fig. 2B and Supplementary Figs. S1–S4). These results suggested the production of new chlorinated pyrroloquinoline analogs by *S. uncialis*, and we then performed a large-scale fermentation of *S. uncialis* SB18002 (10 l) and isolated four major products from the fermentation broth for structural characterization. They were identified as AME A (**21**), B (**22**), and C (**23**), named after the characteristic C-4a methyl ester functional group unprecedented in the AMM scaffold known to date, as well as a new ammosamaic acid congener (**24**) (Fig. 2C), by extensive spectroscopic analysis and direct comparison of their NMR data (Table 1 and Supplementary Figs. S1–S4) with those reported for AMMs (Hughes & Fenical, 2010; Hughes, MacMillan, Gaudêncio, Jensen, et al., 2009; Reddy et al., 2010; Williams et al., 2008).

AME B (**22**) was isolated as purple powder. It was readily identified as a methyl ester variant of AMM B (**2**) (Fig. 2C) through direct comparison of the NMR and HR-ESI-MS data (Table 1 and Supplementary Fig. S2) (Reddy et al., 2010).

AME C (**23**) was isolated as purple powder. HR-ESI-MS analysis of **23** afforded an [M + H]^+^ ion at *m/z* 293.0448 (Supplementary Fig. S3E), giving the molecular formula as C_12_H_9_ClN_4_O_3_. The diagnostic ^1^H and ^13^C NMR resonances showed high similarity between **22** and **23**, with the only differences at *N*-1 and its nearest carbon chemical shifts at C-2 and C-8a (Table 1 and 9). Together with the loss of the *N*-CH_3_ signal, **23** was assigned as the *N*-desmethyl congener of **22** (Fig. 2C), the structure of which was further supported by extensive 2D NMR analysis, as exemplified, in particular, by the clear HMBC correlations from 1-NH to C-2a and C-5b (Supplementary Figs. S3 and S5).

The new ammosamaic acid congener (**24**) was isolated as purple powder. The molecular formula of **24** was established as C_12_H_9_ClN_4_O_3_ by HR-ESI-MS ([M + H]^+^ ion at *m/z* 293.0444, Supplementary Fig. S4E). With the exception of 14 mass unit less and an extra *N*-CH_3_ signal (*δ*_H_ 3.60, 3H, s; *δ*_C_ 29.1), **24** displayed very similar NMR data to those of the C-2 oxidized ammosamaic acid (**18**) (Jordan & Moore, 2016). Detailed comparison of overall NMR data revealed that the major differences between **24** and **18** resided mainly at *N*-1 and its adjacent carbons C-2 and C-8a, supporting *N*-methylation at *N*-1 for **24** (Table 1). Taken together, **24** was assigned as *N*-methyl and C-2 oxidized ammosamic acid (Fig. 2C), the overall structure of which is fully supported by 2D NMR analysis, including the key HMBC correlations from *N*-CH_3_ (*δ*_H_ 3.60) to C-2 (*δ*_C_ 164.4) and C-8a (*δ*_C_ 107.1) (Supplementary Figs. S4 and S5).

AME A (**21**) was isolated as purplish red solid. HR-ESI-MS analysis of **21** afforded an [M]^+^ ion at *m/z* 291.0632, giving the molecular formula as C_13_H_12_ClN_4_O_2_^+^. The 1D NMR spectra of **21** were very similar to those of **22**. The major differences were that the carbonyl carbon at C-2 (*δ*_C_ 164.1) in **22** was replaced by iminium ion carbon at C-2 (*δ*_H_ 7.95, s; *δ*_C_ 124.3) in **21** (Table 1), leading to the assignment of **21**, in a structural analogy to AMM C (**3**), as an iminium salt (Fig. 2C). The structure of **21** was finally confirmed by 2D NMR analysis, specifically by HMBC correlations from H-2 to C-2a/C-5b/*N*-CH_3_ and from *N*-CH_3_ to C-2/C-8a (Supplementary Figs. S1 and S5) as has been observed previously for **3** (Reimer & Hughes, 2017).

Since it has been demonstrated that AMM B (**2**) is derived from AMM C (**3**) via nonenzymatic oxidation at C-2 (Reimer & Hughes, 2017), the AMEs (**22–24**) are likely also derived from the corresponding iminium ion precursors, such as **21** for **22**, via the same nonenzymatic process (Fig. 3). Although **22** and **23** have been reported previously as synthetic intermediates of **2** (Reddy et al., 2010), we have isolated them for the first time, together with **21** and **24**, as natural products, expanding the structural diversity for the AMM family of natural products.

### Cytotoxicity of the Ammosesters Against Selected Human Cancer Cell Lines

The AMEs (**21–23**), together with the ammosamaic acid congener **24**, were evaluated for their cytotoxic activities against five selected human cancer cell lines, including melanoma (SKMEL-5), breast (MDA-MB-231), central nervous system (SF-295), nonsmall cell lung (NCI-H226), and ovarian (OVCAR-3), with **2** and doxorubicin as controls (Table 2 and Supplementary Fig. S6) (Yan et al., 2016; Yan et al., 2017). Similar to **2, 21** exhibited modest cytotoxicity against all the tested cancer cell lines, with EC_50_ values ranging from 15 to 140 µM, while **22** was active against the SK-MEL-5 and OVCAR-3 cell lines, and **23** and **24** were inactive at the concentrations examined (EC_50_ > 200 µM), suggesting both the *O*-methylation and *N*-methylation contribute to the cytotoxicity. The fact that **21** exhibited an increased cytotoxicity towards the selected human cancer cell lines in comparison to **22–24** is consistent with the previous findings for the AMMs with the pyrroloquinolinium congeners showing the highest cytotoxicity against HCT-116 (Reimer & Hughes, 2017).

### *In Vivo* and *In Vitro* Characterization of Ame24 Confirming Its Function as an *O*-Methyltransferase

The characteristic structural difference between AMEs and AMMs is the presence of a methyl ester or amide at the C-4a of AMEs or AMMs, respectively (Figs. 1 and 2C). Comparative analysis of the *ame* and *amm* BGCs revealed *ame24*, unique to the *ame* BGC and predicted to encode an *O*-methyl transferase, serving as the candidate for *O*-methylation at C-4a in AME biosynthesis (Fig. 2A). To investigate AME biosynthesis, we first carried out *in vivo* studies by inactivating the *ame24* gene in *S. uncialis* SB18002, using the PCR-targeting and λ-RED-mediated mutagenesis method (Gust et al., 2003; Kieser et al., 2000). The genotype of the resultant Δ*ame24* mutant strain *S. uncialis* SB18003 was confirmed by Southern analysis (Supplementary Fig. S7). When cultured under the same condition using *S. uncialis* SB18002 as a control, *S. uncialis* SB18003 failed to produce **21**–**23** but still produced the free acid congener **24** (Supplementary Fig. S7). The latter phenotype would be consistent with Ame24 as an *O*-methyl transferase catalyzing *O*-methylation of a free acid precursor in AME biosynthesis. We next expressed *ame24* in *E. coli* and purified the overproduced Ame24 to homogeneity to directly confirm its activity *in vitro* (Supplementary Fig. S8). In the presence of *S*-adenosyl methionine (SAM), Ame24 catalyzed time-dependent conversion of **24** into a new product, which was absent in the negative control using boiled Ame24 as a control (Fig. 2B). The identity of the new product as **22** was confirmed by HPLC–MS analysis in comparison with an authentic standard. While these findings unambiguously established Ame24 as an *O*-methyltransferase, catalyzing *O*-methylation at C-4a in AME biosynthesis, the low catalytic efficiency of Ame24 with **24** as a substrate, as exemplified by 6% and 12% conversion after 2 and 18 hr, respectively, under the assay conditions (Supplementary Fig. S8), suggested that **24** may not be the preferred substrate. The latter would be consistent with the isolation of **21** and **23** from *S. uncialis* SB18002 fermentation, indicative of the existence of alternative free acids as Ame24 substrates, such as **26** and **17**, respectively (Fig. 3B). All attempts to generate **26** by hydrolysis of **21**, however, failed due to its intrinsic instability, and we also failed to detect **17** in *S. uncialis* SB18002 fermentation, both of which prevented us from directly determining the preferred substrate for Ame24 and thereby the timing of Ame24 catalysis in AME biosynthesis.

### A Unified Pathway for AME and AMM Biosynthesis Diverging from a Common Intermediate with Pathway-Specific Tailoring Enzymes

The similarity in both structures and BGCs suggests a common pathway for AME and AMM biosynthesis (Fig. 3). Thus, inspired by the emerging paradigm for amino acid-derived natural product biosynthesis featuring a scaffold peptide, we propose that AME and AMM biosynthesis shares a unified pathway, diverging from a common intermediate, that is, the nascent product derived upon proteolytic removal of the scaffold peptide (Ting et al., 2019). Such an intermediate would feature a free carboxylic acid, in agreement with **17** (Fig. 3A), which has been isolated from the *Streptomyces* sp. CNR-698 Δ*amm4* mutant strain (Jordan & Moore, 2016). However, *amm4*, predicted to encode an F420-dependent oxidase, is unlikely to be involved in the formation of the C-4a amide for the AMMs as proposed previously (Jordan & Moore, 2016) on the basis of both the presence of *ame5*, an *amm4* homolog, and the absence of an *amm20* homolog in the *ame* BGC (Fig. 2A). Instead, we propose that *amm20*, predicted to encode an amide synthetase, catalyzes C-4a amidation of the common intermediate **17** to afford **19**, which is finally *N*-methylated by Amm23 to yield **3**, completing AMM biosynthesis (Fig. 3C). In a biosynthetic analogy, C-4a *O*-methylation and *N*-methylation of **17** by Ame24 and Ame22, respectively, affording **21**, would account for AME biosynthesis (Fig. 3B).

Finally, we carried out a time course analysis of the AME metabolite profile of *S. uncialis* SB18002, in an attempt to determine the timing of the Ame24-catalyzed *O*-methylation and Ame22-catalyzed *N*-methylation in AME biosynthesis (Supplementary Fig. S9). AME production could be readily observed as early as 4 days of growth, with **22** as the dominant metabolite. After 6 days of fermentation, **21**, the precursor to the nonenzymatically C-2 oxidized product **22**, was observed as the dominant metabolite, supporting **21** as the bona fide final natural product of the AME biosynthetic machinery (Fig. 3B). As fermentation continued, **21** started to decrease with a concomitant increase of **22**, as well as the detection of **23** and **24**. Since **23** and **24** could be viewed as nonenzymatically C-2 oxidized products of intermediates **25** and **26**, respectively, en route to **21**, the fact that **23** and **24** increased with a relatively constant ratio would suggest that *N*-methylation and *O*-methylation of **17**–**21** proceed in two parallel pathways, most likely due to substrate promiscuity of both Ame22 and Ame24 (Fig. 3B).

In conclusion, by mining the genome of *S. uncialis*, we have identified the *ame* BGC that is highly homologous in both gene content and genetic organization to the *amm* BGC and have discovered a sub-family of new AMM congeners, named AMEs (Fig. 2). The AMEs feature a C-4a methyl ester, differing from the C-4a amide functional group characteristic to AMMs, and exhibit modest cytotoxicity against a broad spectrum of human cancer cell lines, expanding both the structure and SAR for the pyrroloquinoline family of natural products (Figs. 1 and 2C, and Table 2). Comparative analysis of the *amm* and *ame* BGCs, together with *in vivo* and *in vitro* studies of *ame24*, support the use of a scaffold peptide as an emerging paradigm for the biosynthesis of the pyrroloquinoline family of natural products. AME and AMM biosynthesis diverges from a common intermediate by evolving the pathway-specific Ame24 *O*-methyltransferase and Amm20 amide synthetase, respectively (Fig. 3). Although the exact timing for each of the steps will have to be determined by additional *in vivo* and *in vitro* experiments in the future, the findings from the current study showcase once again Nature's ingenuity to generate diverse natural products by evolving biosynthetic machineries in a combinatorial fashion.

## Supplementary Material

kuab027_Supplemental_File

## Data Availability

All data generated or analysed during this study are included in this published article and its supplementary material.
