# Supplementary material for: Discovery of ammosesters by mining the Streptomyces uncialis DCA2648 genome revealing new insight into ammosamide biosynthesis
Source: J Ind Microbiol Biotechnol. 2021 Mar 24;48(3-4):kuab027. doi: 10.1093/jimb/kuab027 (PMC8210675; doi:10.1093/jimb/kuab027)
Supplement: kuab027_Supplemental_File [file kuab027_Supplemental_File.pdf]

Discovery of Ammosesters by Mining the *Streptomyces uncialis* DCA2648 Genome Revealing New Insight into Ammosamide Biosynthesis

Jun Luo,[1] Dong Yang,[1,2] Hindra,[1] Ajeeth Adhikari,[1,3] Liao-Bin Dong,[1] Fei Ye,[1] Xiaohui Yan,[1] Christoph Rader,[3] and Ben Shen*,[1,2,4]

[1]Department of Chemistry, The Scripps Research Institute, Jupiter, Florida 33458, United States
[2]Natural Products Discovery Center at Scripps Research, The Scripps Research Institute, Jupiter, Florida 33458, United States
[3]Department of Immunology and Microbiology, The Scripps Research Institute, Jupiter, Florida 33458, United States
[4]Department of Molecular Medicine, The Scripps Research Institute, Jupiter, Florida 33458, United States

Jun Luo, Dong Yang, and Hindra contributed equally to this work.

*Correspondence to: E-mail: shenb@scripps.edu; Tel: (561) 228-2456; Fax: (561) 228-2472

Supplementary Information

**Table S1**	Bacterial strains and plasmids used in this study	S2
**Table S2**	Oligonucleotides used in this study	S2
**Table S3**	Predicted functions of ORFs in the *ame* BGC from *S. uncialis* DCA2648 in comparison with the *amm* BGC from *S.* sp. CNR-698	S3
**Figure S1**	NMR, HR-ESI-MS, and UV spectra of ammosester A (**21**)	S4-S6
**Figure S2**	NMR, HR-ESI-MS, and UV spectra of ammosester B (**22**)	S7-S9
**Figure S3**	NMR, HR-ESI-MS, and UV spectra of ammosester C (**23**)	S10-S12
**Figure S4**	NMR, HR-ESI-MS, and UV spectra of ammosamaic acid congener (**24**)	S13-S15
**Figure S5**	HMBC correlations of **21-24**	S16
**Figure S6**	Cytotoxicity assay of **21-24** in comparison with **2** and doxorubicin	S17
**Figure S7**	Inactivation of *ame24* by gene replacement	S18
**Figure S8**	SDS-PAGE analysis and *in vitro* assay of Ame24	S19
**Figure S9**	HPLC analysis of metabolite profiles following the time courses of *S. uncialis* SB18002 fermentation	S20
**References**		S21

Table S1. Bacterial strains and plasmids used in this study

Strain	Genotype and description	Reference/Source
E. coli DH5α	*E. coli* host for plasmid construction	Sambrook et al., 2001/Life Technologies
E. coli ET12567/pUZ8002	Methylation-deficient *E. coli* host for intergeneric conjugation; contains pUZ8002, a non-transmissible *oriT* mobilizing plasmid	Kieser et al., 2000
E. coli BW25113/pIJ790	*E. coli* host for PCR targeting	Gust et al., 2003
E. coli BL21 (DE3)	*E. coli* host for protein production	Life Technologies
S. uncialis DCA2648	wild-type	Davies et al., 2005
S. uncialis SB18002	*S. uncialis* with *ΔclaO-D::*scar, cladoniamide production abolished	This study
S. uncialis SB18003	The Δ*ame24* mutant of SB18002, *ame24* replaced by apramycin resistance gene *aac(3)IV*	This study
**Plasmid/Cosmid**		
pIJ773	Plasmid containing the apramycin resistance cassette (*aac(3)IV* + *oriT*)	Gust et al., 2003
pBS18005	Cosmid 13F4 from *S. uncialis* cosmid library, containing partial *ame* BGC	This study
pBS18006	pBS18005 with *ame24* inactivated with *aac(3)IV* + *oriT* by PCR targeting (i.e., Δ*ame24*)	This study
pBS3080	pRSFDuet-1 derived plasmid containing a BsmFI site for ligation-independent cloning (LIC) and encodes a TEV cleavage site	Lohman et al., 2013
pBS18007	Plasmid for *ame24* expression in *E. coli* BL21 (DE3)	This study

Table S2. Oligonucleotides used in this study

Oligonucleotide	Nucleotide Sequence (5'-3')	Function
KOame24-F	GTGGAGAGCTCACCCCGCGTCGCCCCGGAACACCACG GGGGATTCCGGGGATCCGTCGA	Gene replacement
KOame24-R	GTGAGTGTGCGGTTAACGACTTGTGGAACGGGGCTTGA CACCTG TAGGCTGGAGCTGCTT	Gene replacement
SA-ame24-F	CAGCTCGTTCAGCGCGGTC	Southern blot analysis
SA-ame24-R	CTCCATGTTCGCGGGCCAG	Southern blot analysis
GE-ame24-F	AAAACCTCTATTTCCAGTCGGTGGAGAGCTCACCCCG	Gene expression
GE-ame24-R	TACTTACTTAAATGTTATTAACGACTTGTGGAACGGG	Gene expression

Table S3. Predicted functions of ORFs in the *ame* BGC from *S. uncialis* DCA2648 in comparison with the *amm* BGC from *S.* sp. CNR-698 (Jordan & Moore, 2016)

Gene	aa	Putative function	Homologue	% protein ID	Genbank No.
ame1, ame2		Beyond the boundary	*amm1*	Beyond the boundary	
ame3	444	Putative transporter	*amm2*	70	AB852_RS23805
ame4	650	Tryptophan halogenase	*amm3*	75	AB852_RS23810
ame5	145	Putative pyridoxamine oxidase	*amm4*	66	AB852_RS23815
ame6	65	Scaffold peptide	*amm6*	61	AB852_RS39295
ame7	611	Unknown	*amm7*	75	AB852_RS23825
ame8	821	Putative lantibiotic dehydratase	*amm8*	67	AB852_RS23830
ame9	819	Peptide-amino acyl tRNA ligase	*amm9*	69	AB852_RS23835
ame10	508	Unknown	*amm10*	55	AB852_RS23840
ame11	892	Putative lantibiotic dehydratase	*amm11*	60	AB852_RS23845
ame12	438	Putative peptidase	*amm12*	62	AB852_RS23850
ame13	370	Unknown	*amm13*	52	AB852_RS23855
ame14	367	Putative amino acid oxidase	*amm14*	70	AB852_RS23860
ame15	221	Unknown	*amm15*	73	AB852_RS23865
ame16	277	Putative flavoprotein	*amm16*	60	AB852_RS38760
ame17	217	Putative flavin reductase	*amm17*	71	AB852_RS23875
ame18	831	Putative lantibiotic dehydratase	*amm18*	63	AB852_RS38765
ame19	436	Putative peptidase	*amm19*	75	AB852_RS23885
			amm20*	**Amide synthetase**	
ame20	255	Unknown	*amm25*	60	AB852_RS23890
ame21	189	Putative transcription factor	*amm24*	76	AB852_RS23895
ame22	340	*N*-methyltransferase	*amm23*	60	AB852_RS23900
ame23	532	Unknown	*amm22*	57	AB852_RS23905
ame24*	**286**	***O*-methyltransferase**			AB852_RS23910
ame25, ame26		Beyond the boundary	*amm26, amm27*	Beyond the boundary	

*The pathway specific *ame24* (colored blue), the deduced product of which shows 29% amino acid identity to the Aave_2207 (A1TP97.1) *O*-methyltransferase, is annotated to encode an *O*-methyltransferase for AME biosynthesis, which is confirmed experimentally in this study.
**The pathway specific *amm20* (colored red), the deduced product of which shows 41% amino acid identity to the AsnO (O05272.3) amide synthetase, is re-annotated to encode an amide synthetase for AMM biosynthesis on the basis of comparative analysis of the *ame* and *amm* BGCs in this study.

Figure S1A. ^{1}H NMR spectrum of ammosester A (**21**) (700 MHz, DMSO-d_6)

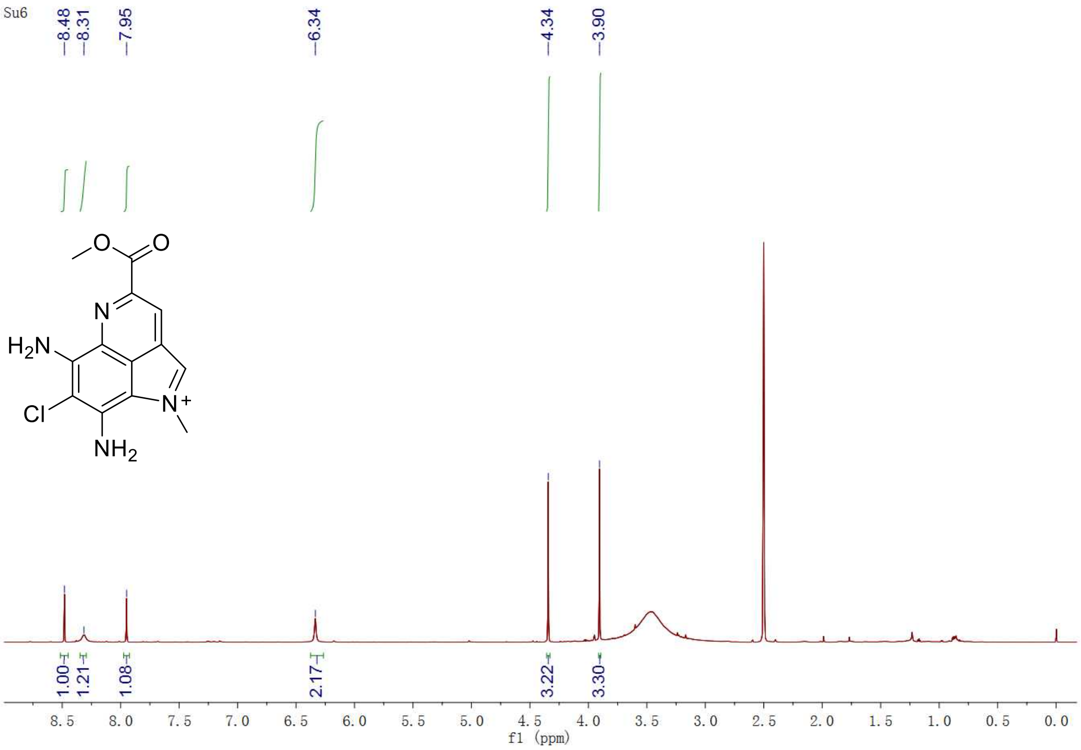

Figure S1B. ^{13}C NMR spectrum of ammosester A (**21**) (175 MHz, DMSO-d_6)

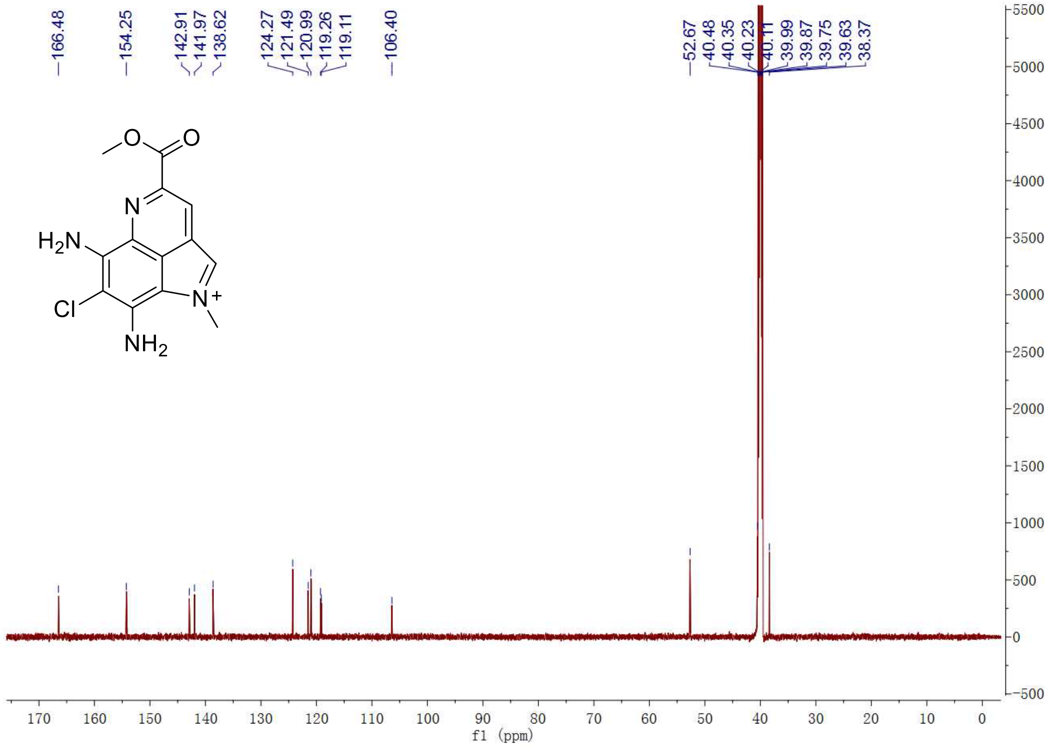

Figure S1C. HSQC spectrum of ammosester A (**21**) (DMSO-d_6)

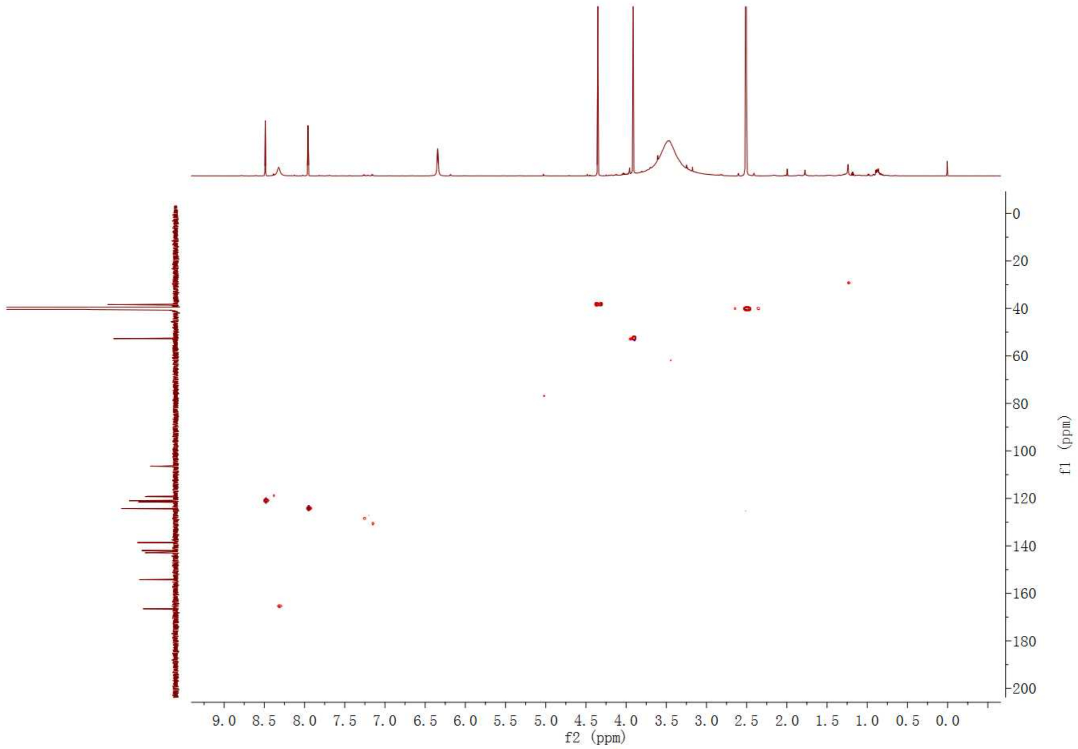

Figure S1D. HMBC spectrum of ammosester A (**21**) (DMSO-d_6)

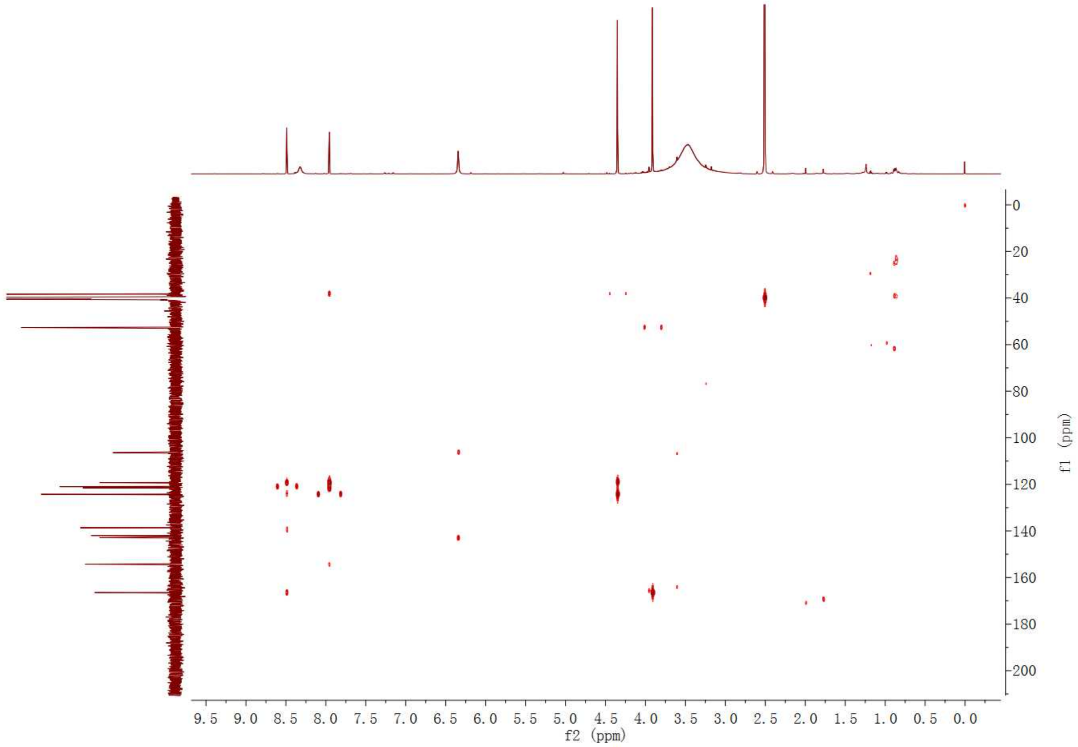

Figure S1E. HR-ESI-MS spectrum of ammosester A (**21**)

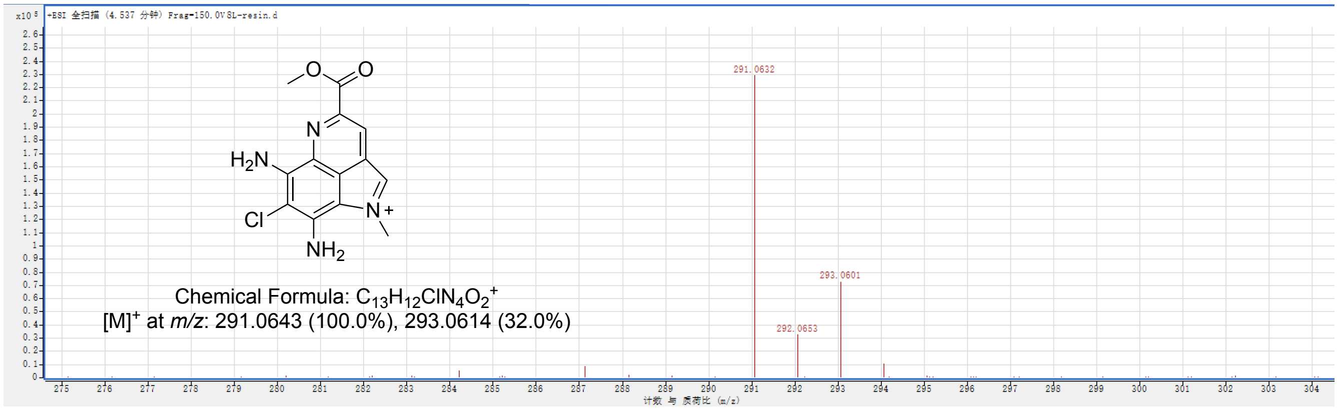

Figure S1F. UV spectrum of ammosester A (**21**)

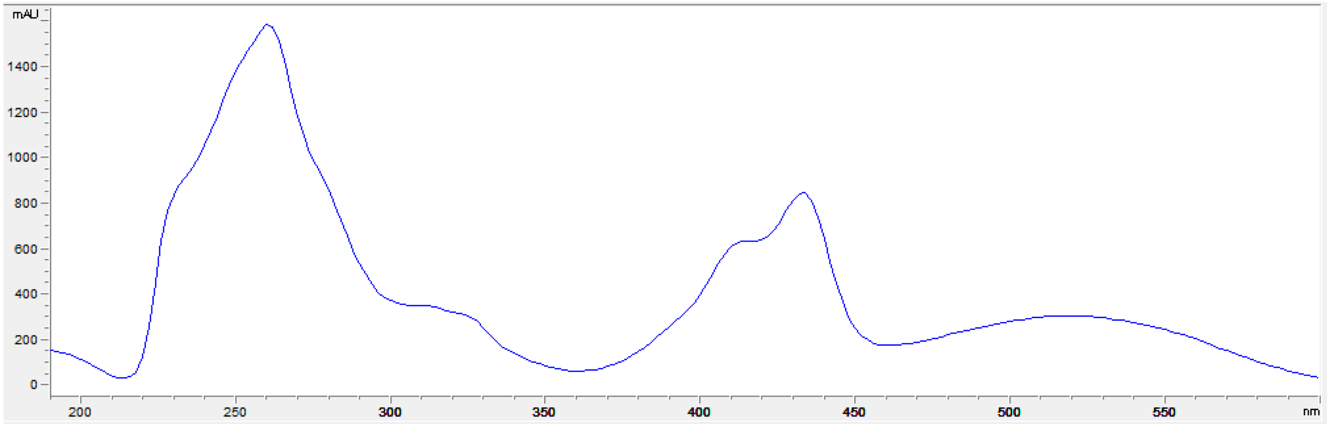

Figure S2A. ^{1}H NMR spectrum of ammosester B (**22**) (700 MHz, DMSO-d_6)

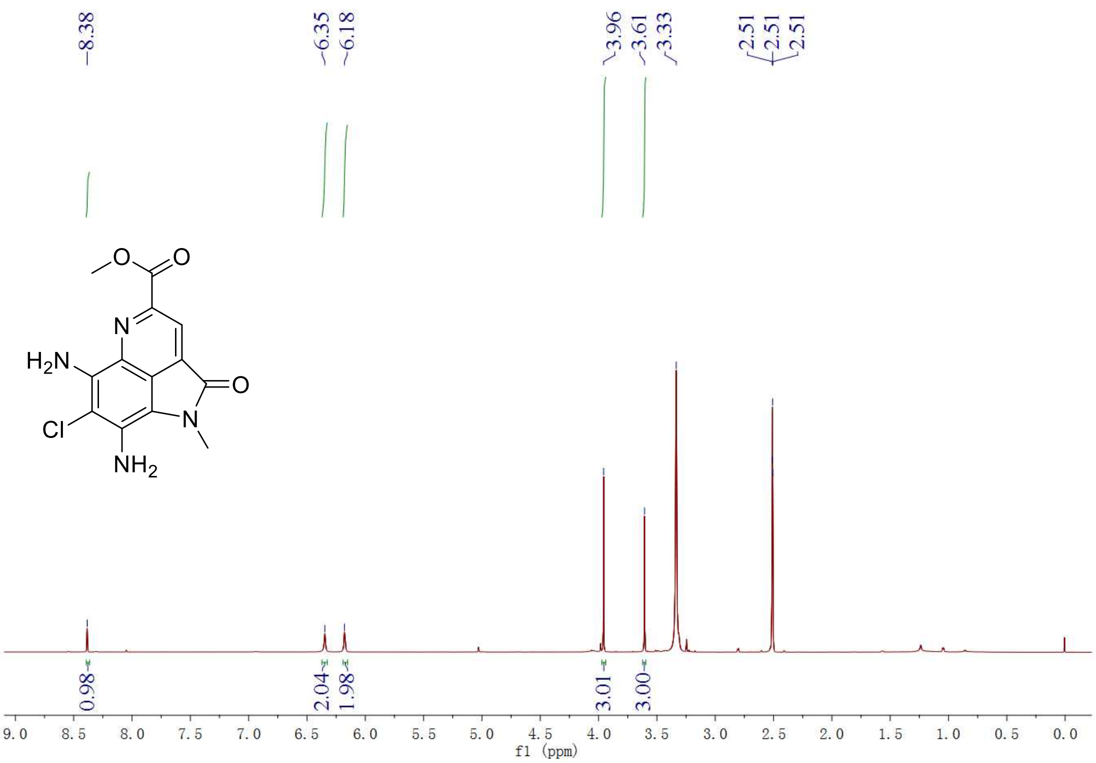

Figure S2B. ^{13}C NMR spectrum of ammosester B (**22**) (175 MHz, DMSO-d_6)

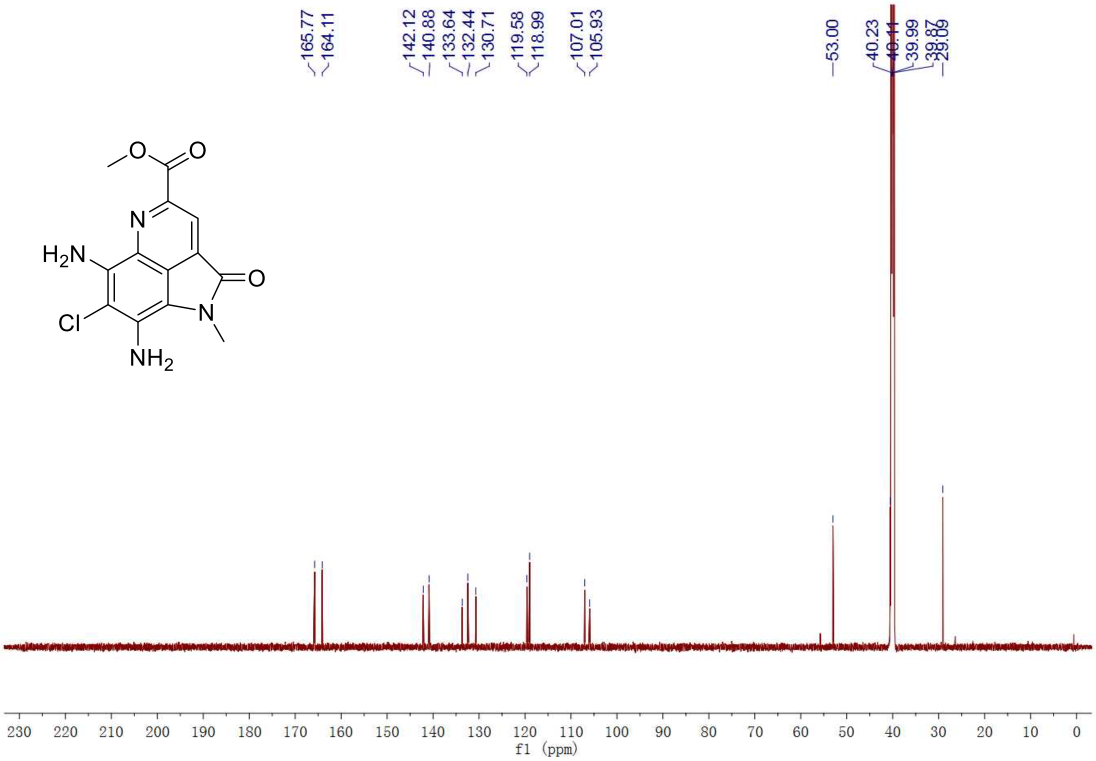

Figure S2C. HSQC spectrum of ammosester B (**22**) (DMSO-d_6)

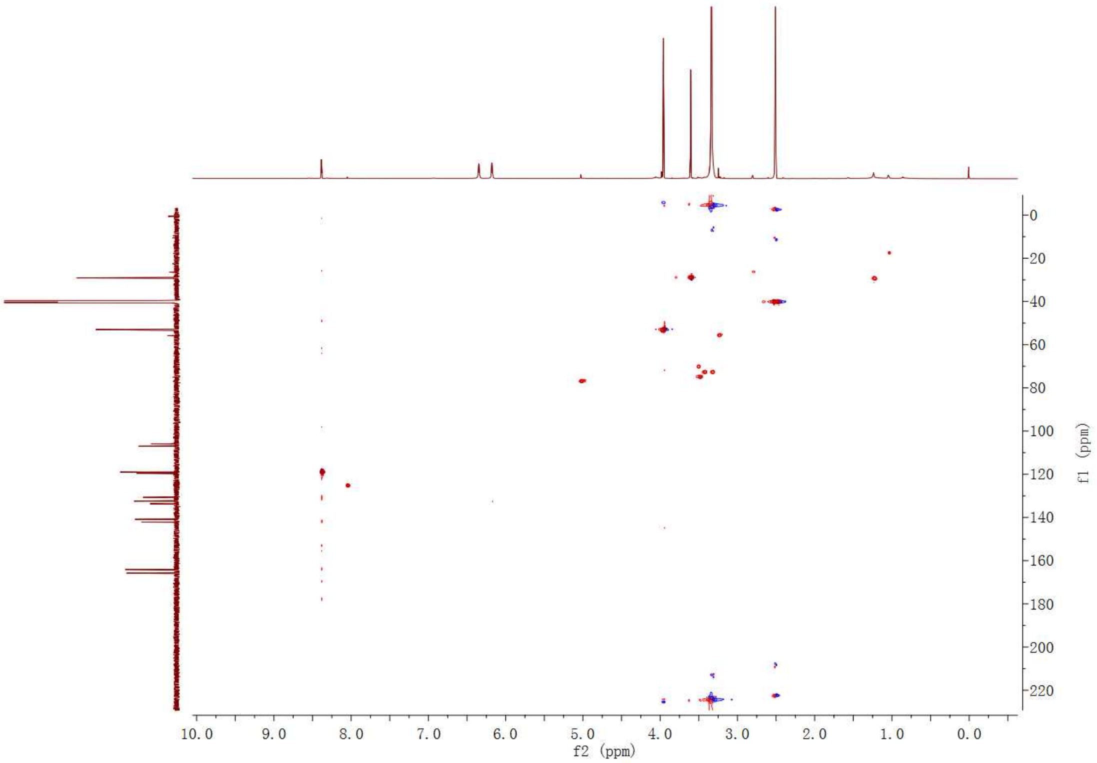

Figure S2D. HMBC spectrum of ammosester B (**22**) (DMSO-d_6)

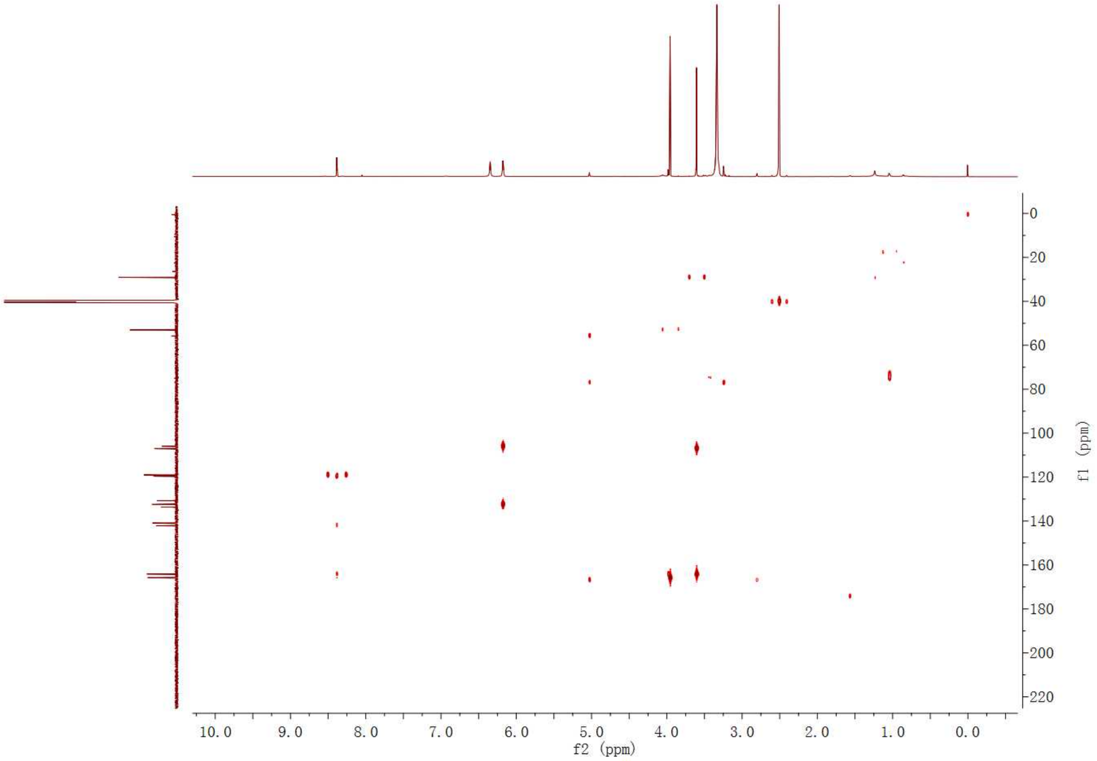

Figure S2E. HR-ESI-MS spectrum of ammosester B (**22**)

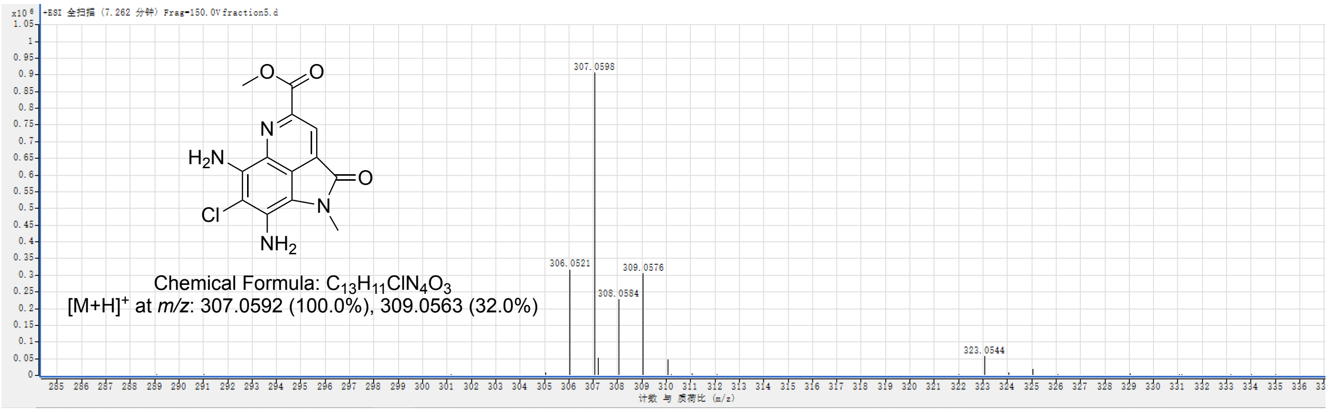

Figure S2F. UV spectrum of ammosester B (**22**)

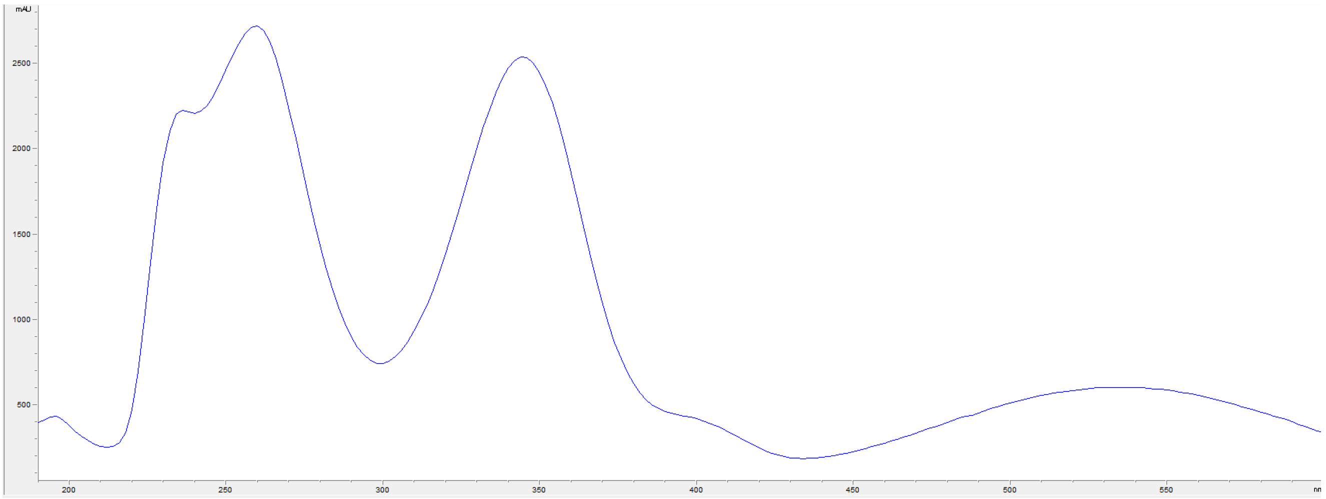

Figure S3A. ^{1}H NMR spectrum of ammosester C (**23**) (700 MHz, DMSO-d_6)

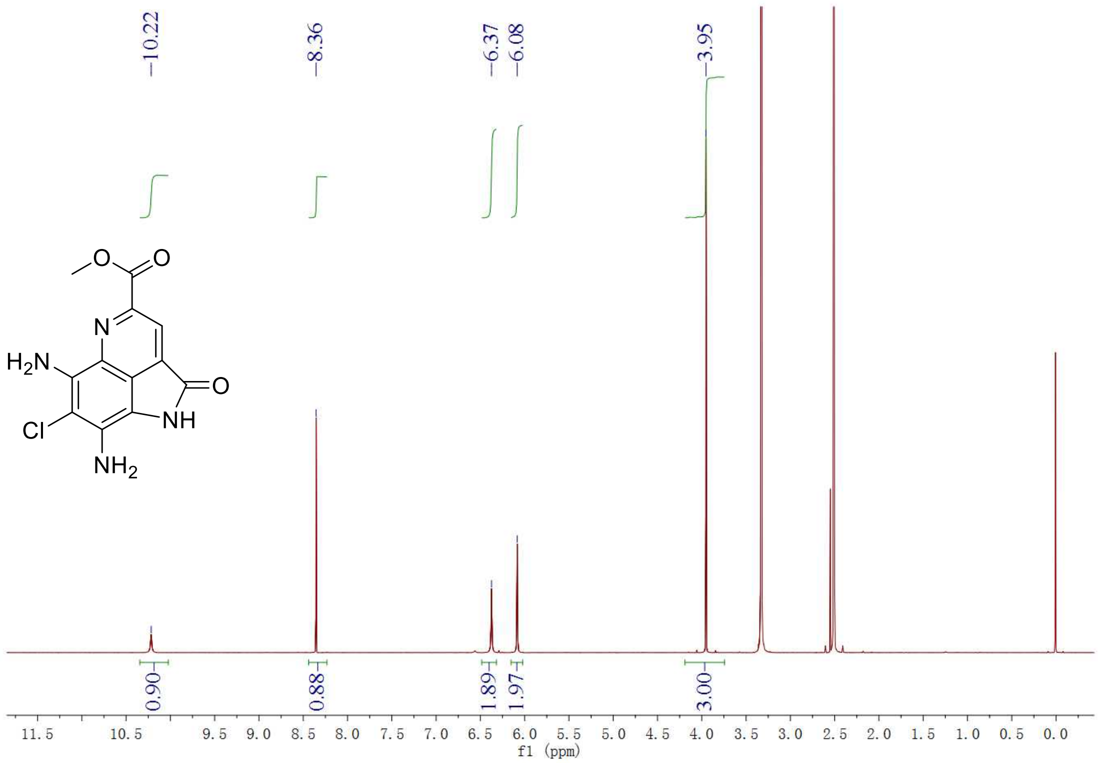

Figure S3B. ^{13}C NMR spectrum of ammosester C (**23**) (175 MHz, DMSO-d_6)

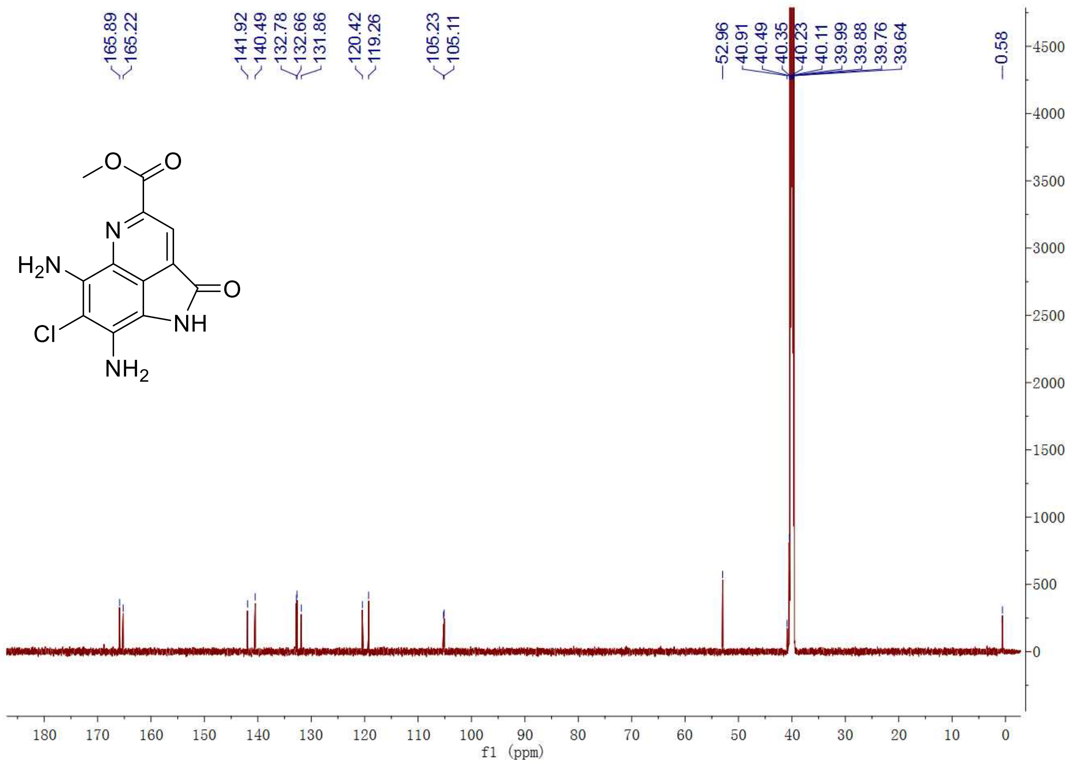

Figure S3C. HSQC spectrum of ammosester C (**23**) (DMSO-d_6)

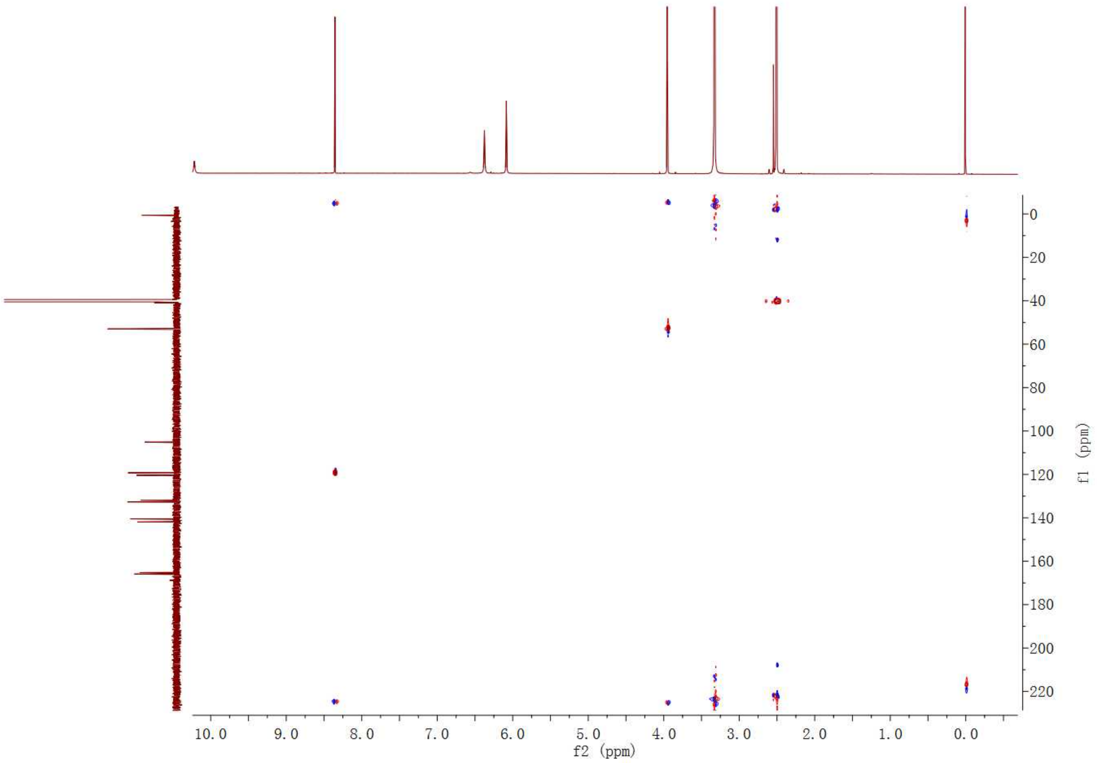

Figure S3D. HMBC spectrum of ammosester C (**23**) (DMSO-d_6)

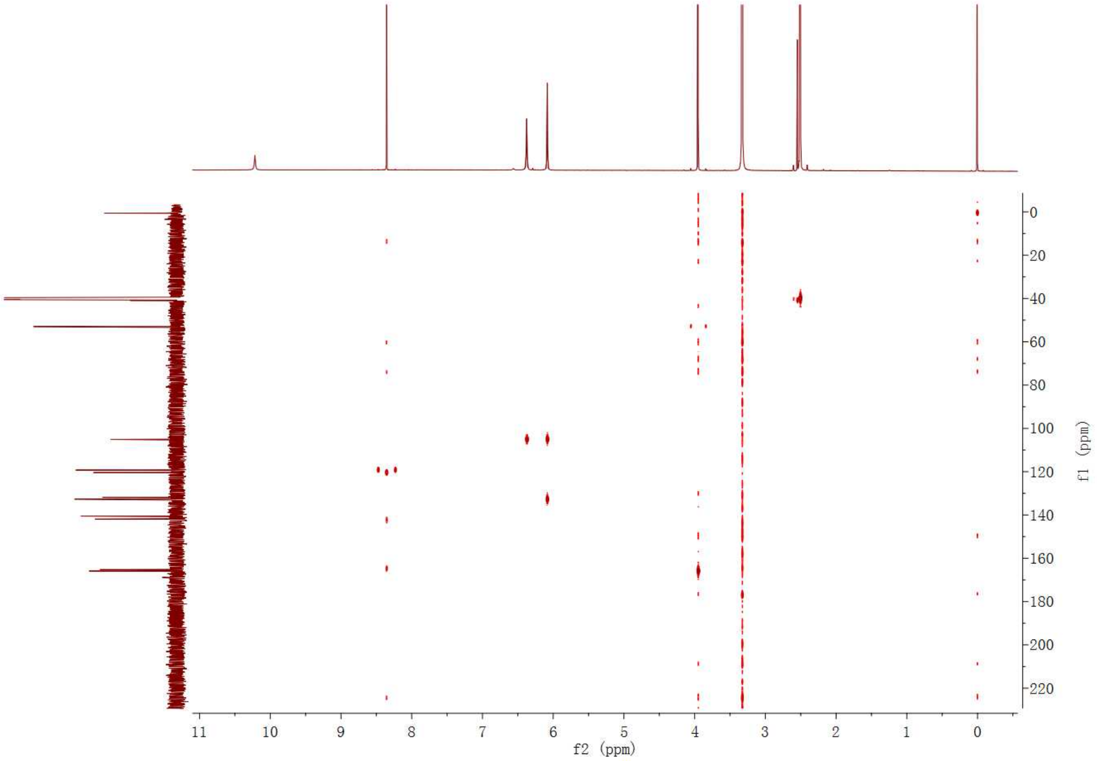

Figure S3E. HR-ESI-MS spectrum of ammosester C (**23**)

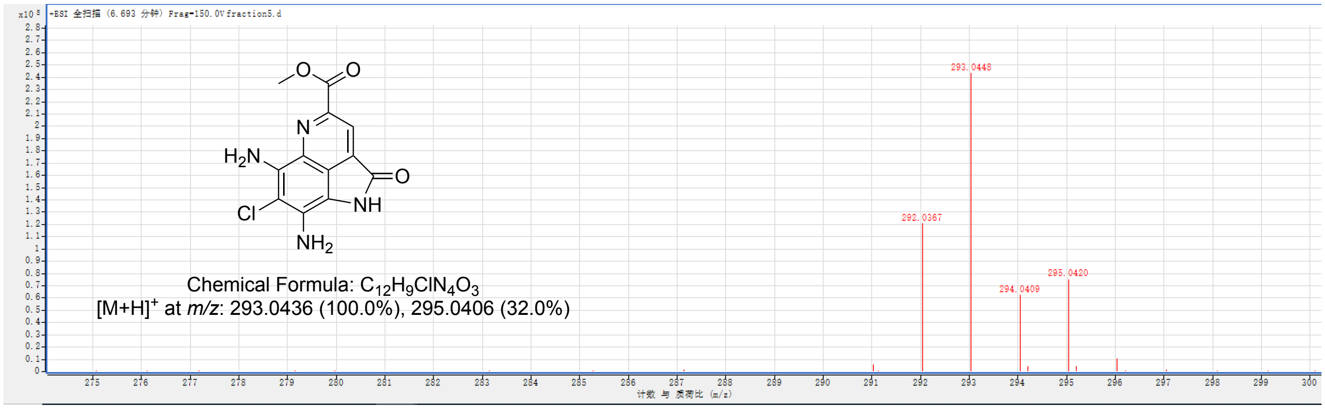

Figure S3F. UV spectrum of ammosester C (**23**)

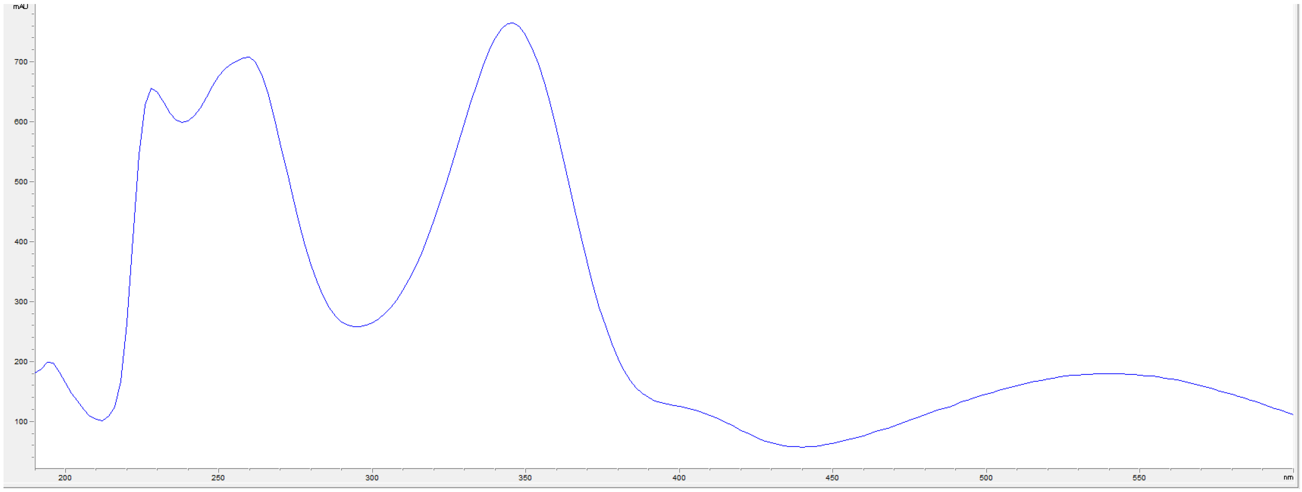

Figure S4A. ^{1}H NMR spectrum of the ammosamaic acid congener (**24**) (700 MHz, DMSO-d_6)

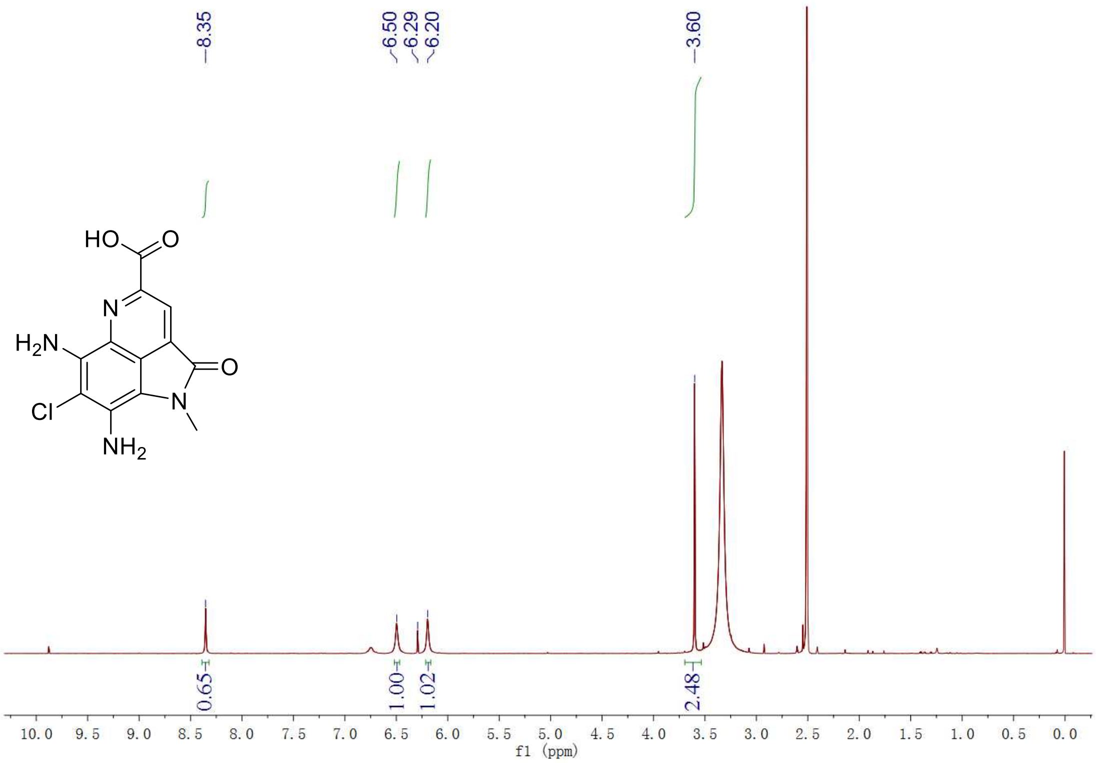

Figure S4B. ^{13}C NMR spectrum of the ammosamaic acid congener (**24**) (175 MHz, DMSO-d_6)

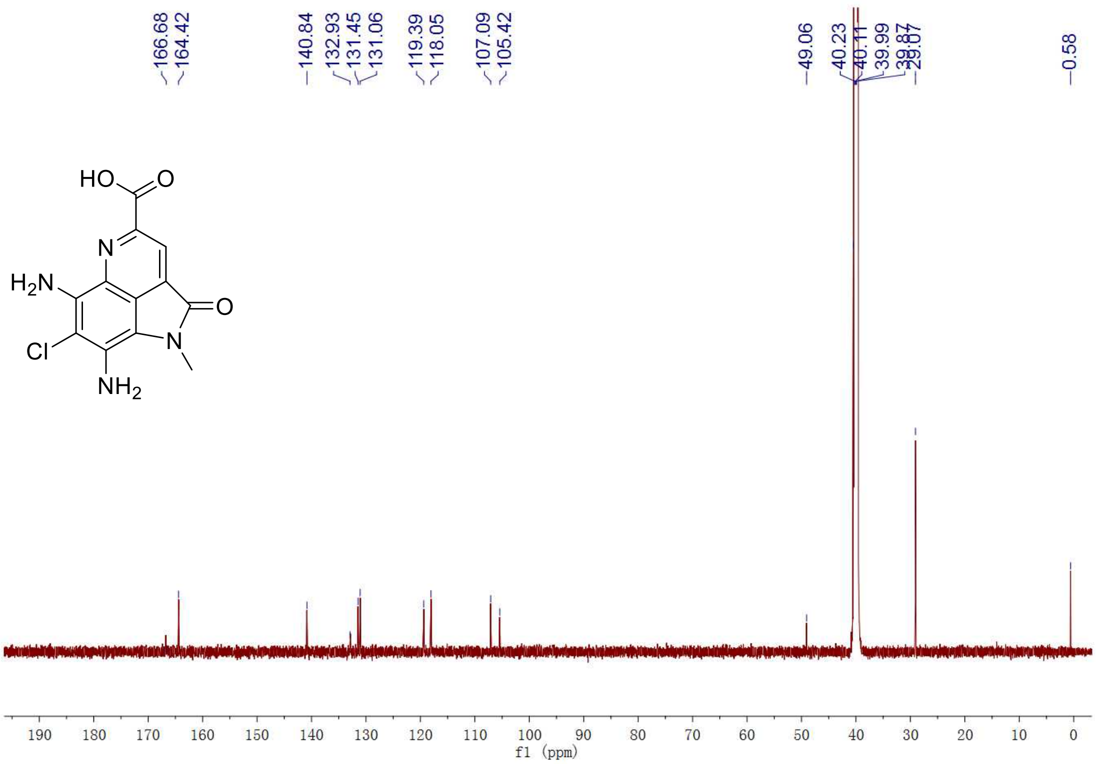

Figure S4C. HSQC spectrum of the ammosamaic acid congener (**24**) (DMSO-d_6)

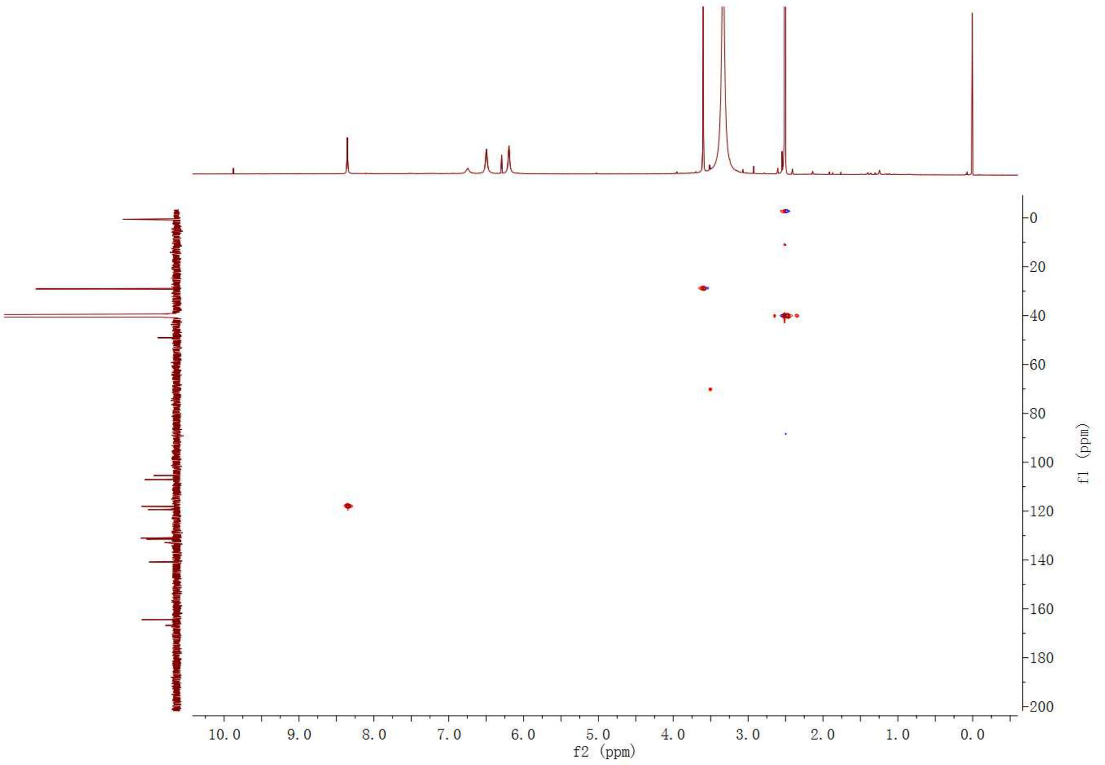

Figure S4D. HMBC spectrum of the ammosamaic acid congener (**24**) (DMSO-d_6)

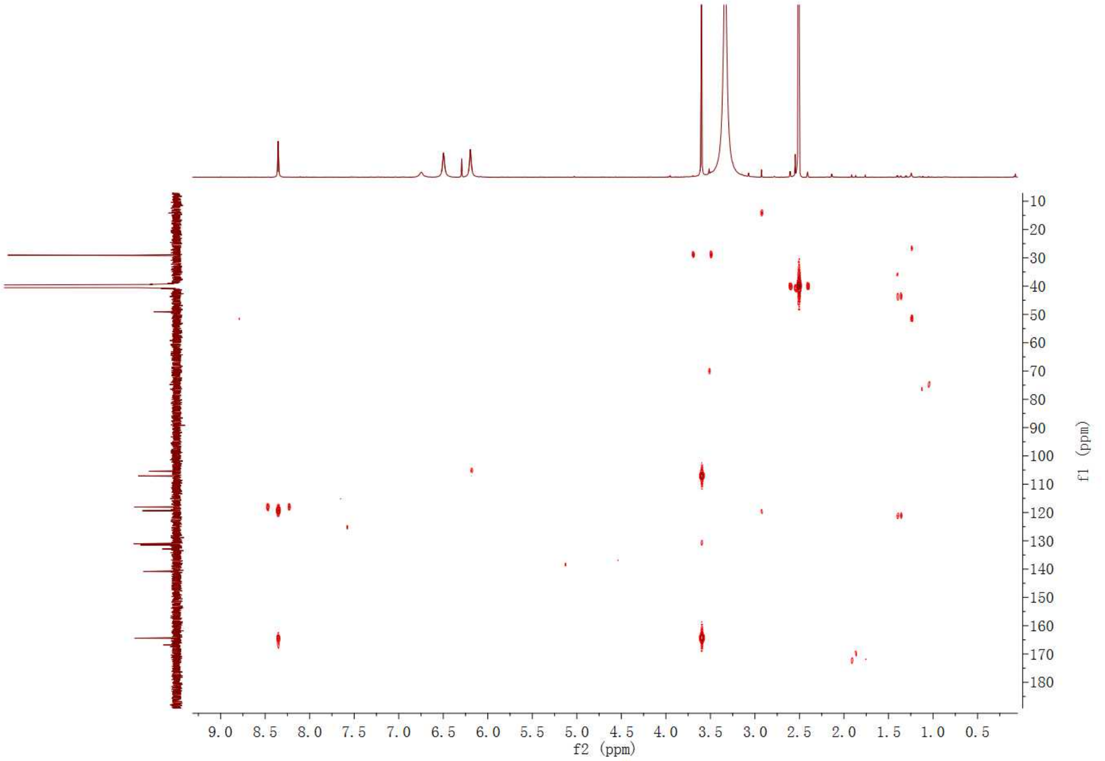

Figure S4E. HR-ESI-MS spectrum of the ammosamaic acid congener (**24**)

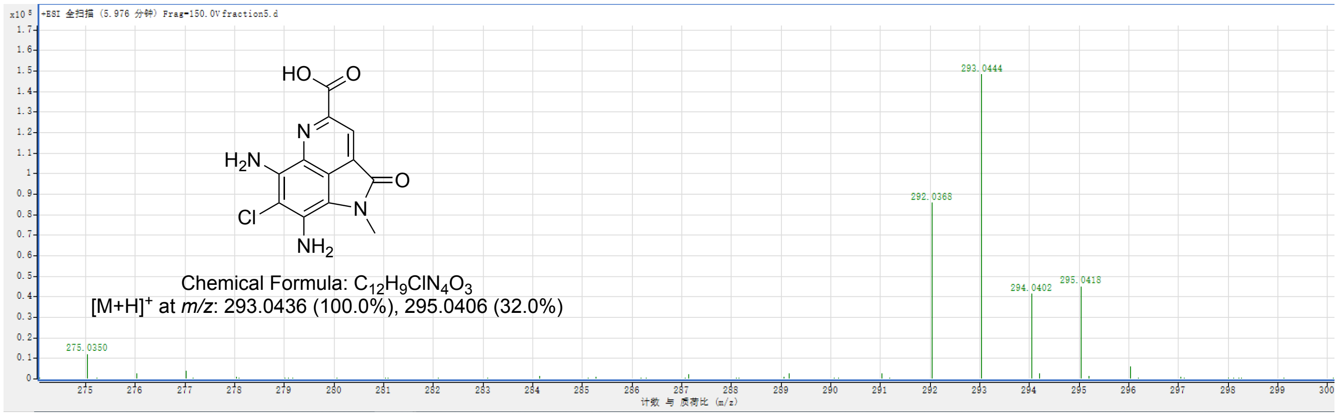

Figure S4F. UV spectrum of the ammosamaic acid congener (**24**)

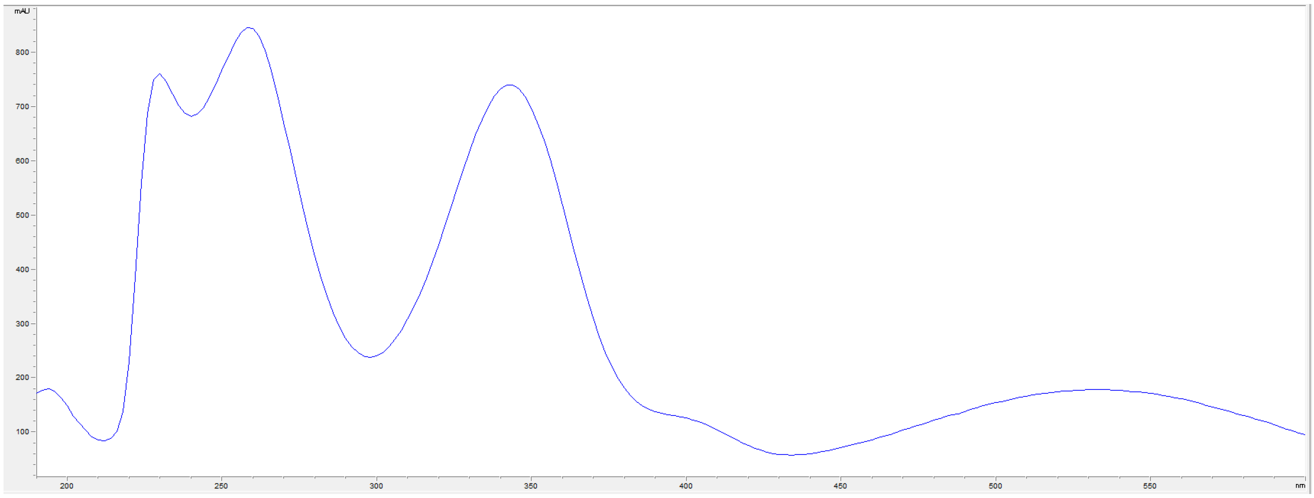

Figure S5. HMBC correlations of ammosester A (**21**), B (**22**), C (**23**), and the ammosamaic acid congener (**24**)

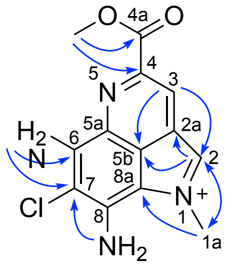

Ammosester A (**21**)

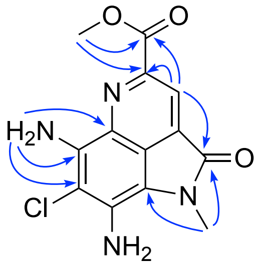

Ammosester B (**22**)

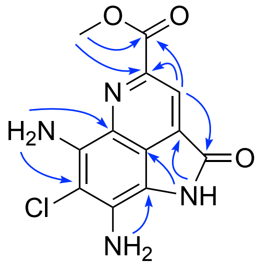

Ammosester C (**23**)

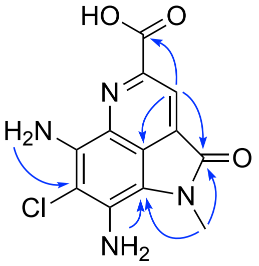

Ammosamaic acid congener (**24**)

Figure S6. Cytotoxicity assay of **21-24** in comparison with ammosamide B (**2**) and doxorubicin

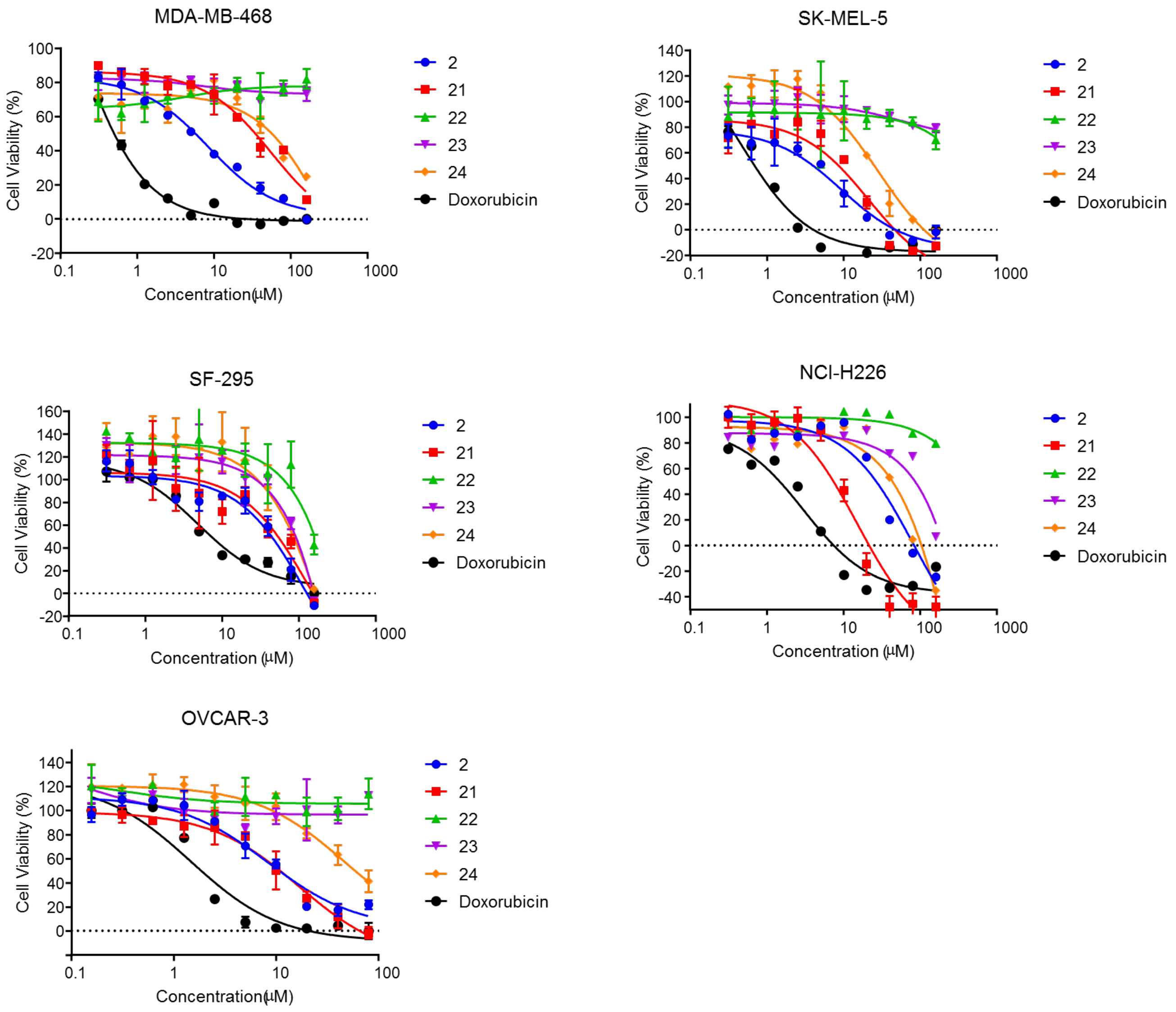

Figure S7. Inactivation of *ame24* by gene replacement. (A) Construction of the Δ*ame24* gene replacement mutant strain *S. uncialis* SB18003 via a double crossover homologous recombination event in *S. uncialis* SB18002. (B) Southern analysis of the genomic DNAs isolated from SB18002 and SB18003 strains digested with FspI and BamHI and hybridized with a 574-bp PCR-amplified probe, showing the expected sizes of 6.5-kb for SB18002 and 1.4-kb for the SB18003, respectively. Lane 1, DNA Molecular Weight Marker VII (Roche); lane 2, SB18002; lane 3, SB18003 (Δ*ame24* mutant). (C) HPLC chromatograms of metabolite profiles from SB18002 and SB18003 (Δ*ame24* mutant).

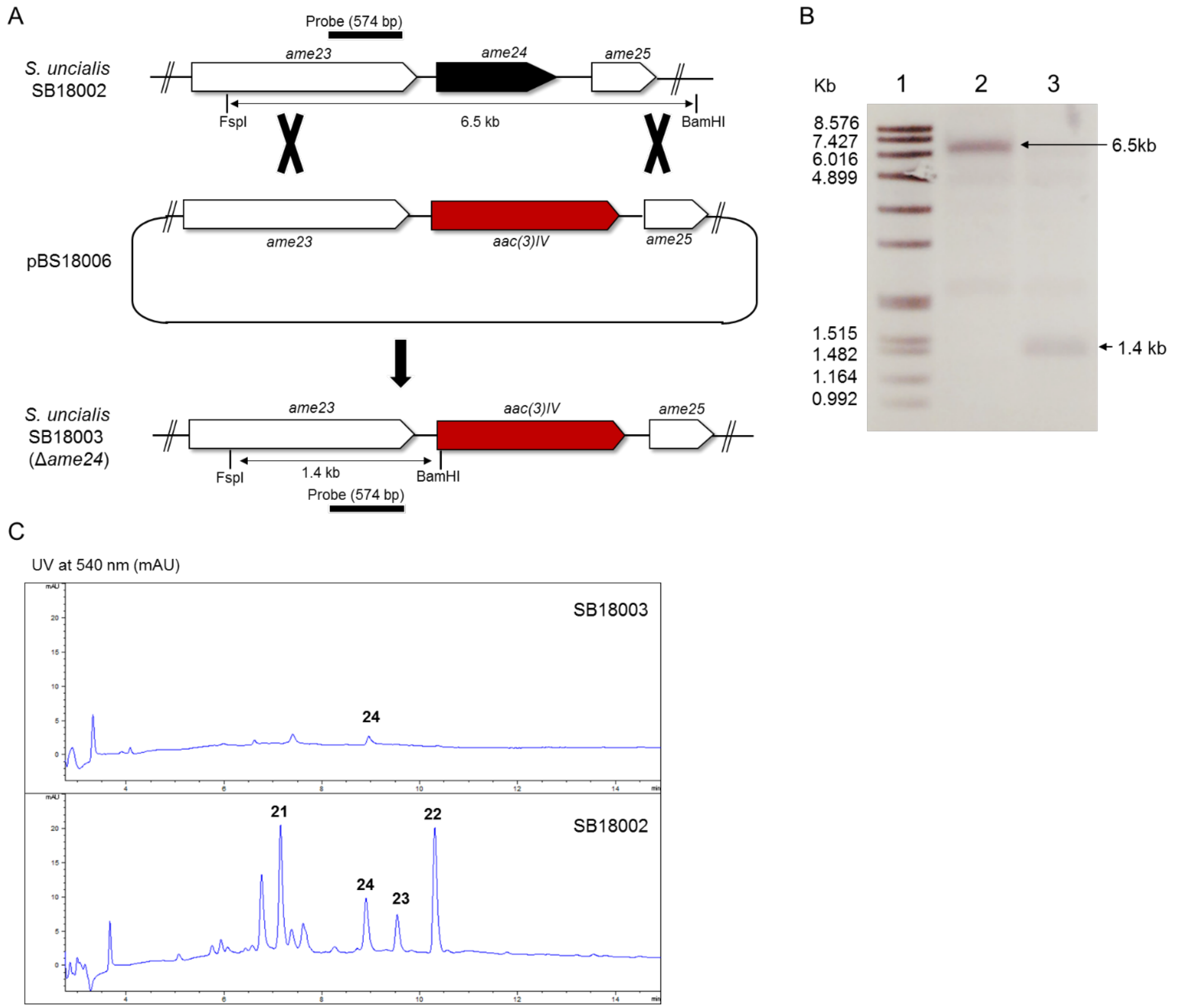

Figure S8. SDS-PAGE analysis and in vitro assay of Ame24. (A) SDS-PAGE gel of purified Ame24. Lane 1, Unstained Protein Ladder (NEB); lane 2, purified N-His$_6$-Ame24 (309 amino acids, ~33.6 kDa). (B) *In vitro* assays of Ame24 with ammosamaic acid congener (**24**) and SAM as substrates. (C) HPLC profiles at different reaction times and calculated yields based on the peak areas of product **22**.

A

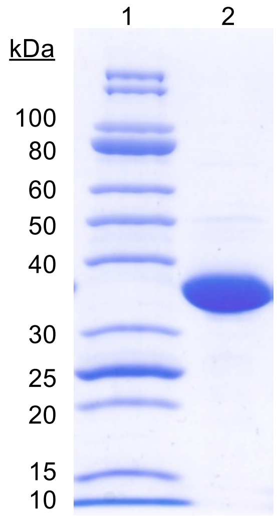

B

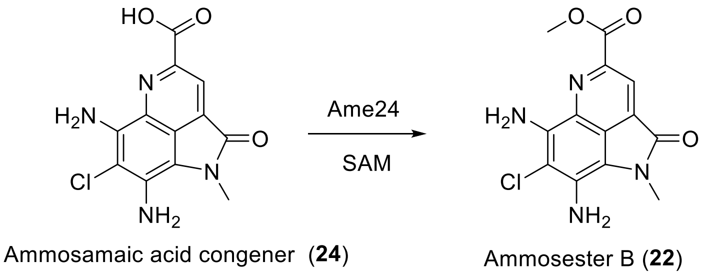

C

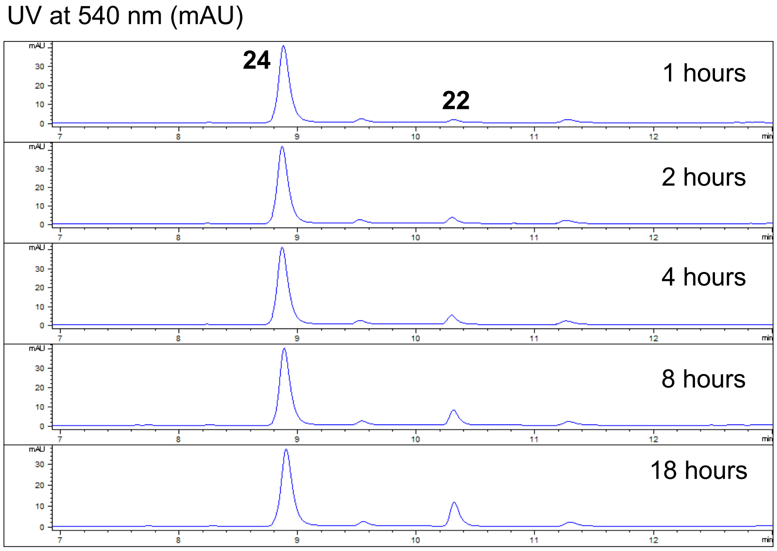

Time (hours)	Yield
1	3.3%
2	5.7%
4	8.9%
8	14.3%
18	20.1%

Figure S9. HPLC analysis of metabolite profiles following the time courses of *S. uncialis* SB18002 fermentation

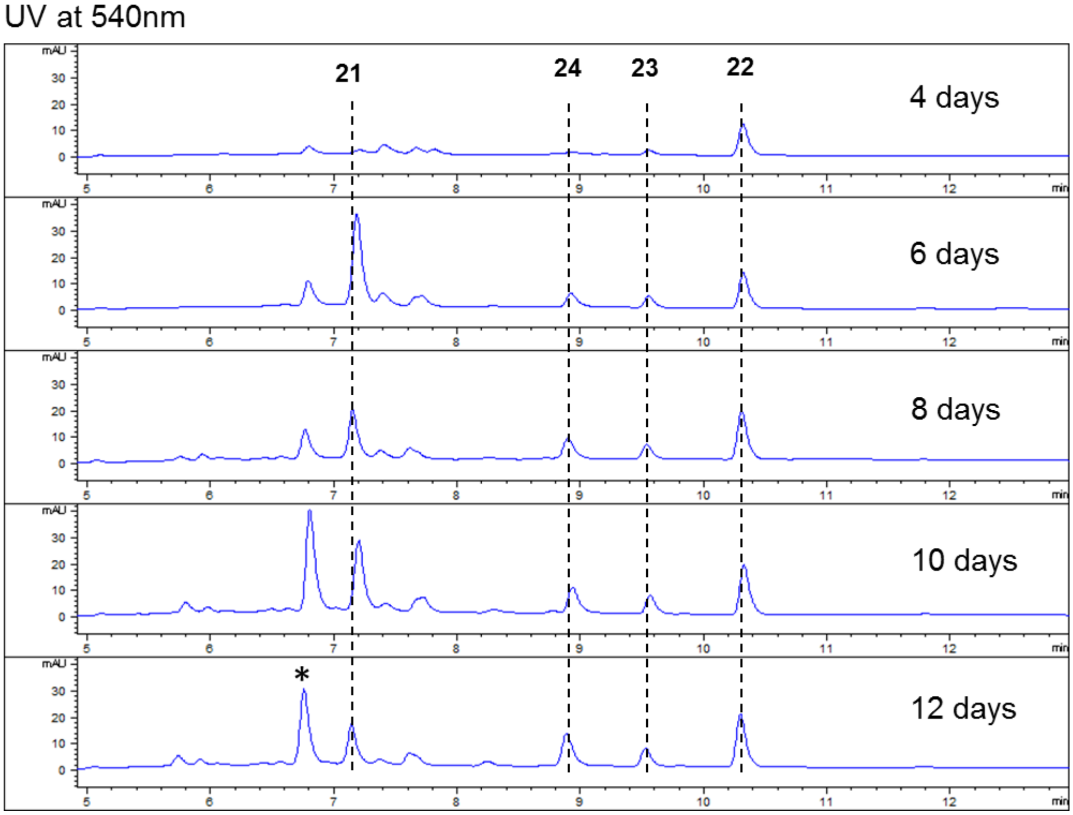

*A red compound; decomposed during purification.

References

Davies, J., Wang, H., Taylor, T., Warabi, K., Huang, X. H., & Andersen, R. J. (2005). Uncialamycin, a new enediyne antibiotic. Organic Letters, 7:5233-5236. https://doi.org/10.1021/ol052081f

Gust, B., Challis, G. L., Fowler, K., Kieser, T., & Chater, K. F. (2003). PCR-targeted *Streptomyces* gene replacement identifies a protein domain needed for biosynthesis of the sesquiterpene soil odor geosmin. Proceedings of the National Academy of Sciences, 100: 1541−1546. https://doi.org/10.1073/pnas.0337542100

Jordan, P. A., & Moore, B. S. (2016). Biosynthetic pathway connects cryptic ribosomally synthesized posttranslationally modified peptide genes with pyrroloquinoline alkaloids. Cell Chemical Biology, 23:1504−1514. https://doi.org/10.1016/j.chembiol.2016.10.009

Kieser, T., Bibb, M. J., Buttner, M. J., Chater, K. F., & Hopwood, D. A. (2000) *Practical Streptomyces Genetics*, The John Innes Foundation: Norwich, U. K.

Lohman, J. R., Bingman, C. A., Phillips Jr, G. N., & Shen, B. (2013). Structure of the bifunctional acyltransferase/decarboxylase LnmK from the leinamycin biosynthetic pathway revealing novel activity for a double-hot-dog fold. Biochemistry, 52:902−911. https://doi.org/10.1021/bi301652y

Sambrook, J., & Russel, D. (2001) *Molecular Cloning: A Laboratory Manual*, 3rd ed., Cold Spring Harbor Laboratory Press: Cold Spring Harbor, NY.
